# Panoramic view of MDH1: driving cancer progression and shaping the tumor immune microenvironment

**DOI:** 10.3389/fimmu.2025.1631449

**Published:** 2025-08-28

**Authors:** Yunchen Lou, Yunwei Lou, Yao Cheng, Beining Xu, Hanbin Chen, Yinwei Dai

**Affiliations:** ^1^ Department of Respiratory, Zhuji Affiliated Hospital of Wenzhou Medical University, Zhuji, Zhejiang, China; ^2^ Department of Oncology, The First Affiliated Hospital of Wenzhou Medical University, Wenzhou, Zhejiang, China; ^3^ Department of Obstetrics and Gynecology, The Second Affiliated Hospital and Yuying Children’s Hospital of Wenzhou Medical University, Wenzhou, Zhejiang, China

**Keywords:** single cell RNA-sequencing, spatial transcriptomics, lung adenocarcinoma, macrophage, glycolysis

## Abstract

**Background:**

Malate dehydrogenase 1 (MDH1), an NAD(H)-dependent isoenzyme, is a key component of the malate-aspartate shuttle (MAS). A significant association has been observed between MDH1 expression and various characteristics of the tumor microenvironment across different cancer types.

**Methods:**

This study provides comprehensive pan-cancer analyses exploring the expression patterns, clinical and pathological correlations, genetic alterations, immunogenomic profiles, single-cell dynamics, alternative splicing signatures, and pharmacological sensitivities related to MDH1. Drug sensitivity profiling and molecular docking techniques have been employed to identify potential anti-cancer compounds targeting MDH1. Experiments have also been conducted to investigate the biological function of MDH1 in lung adenocarcinoma (LUAD) and to confirm the interaction between MDH1 and macrophages using immunofluorescence assays.

**Results:**

MDH1 expression levels are elevated across a wide range of malignancies, and overexpression of MDH1 was consistently linked to poor prognosis in multiple cancer subtypes. Moreover, MDH1 expression shows complex correlations with various immune cell populations, particularly macrophages, and cohort analysis of both bulk and pan-cancer single-cell immunotherapy data suggest that MDH1 could serve as a predictive marker for immunotherapy responses. Moreover, knockdown of MDH1 suppresses macrophage invasion. To investigate the role of MDH1 in LUAD cells, a potential inhibitor of MDH1 was identified, BI-2536, and has been confirmed to impact MDH1 activity and impede the growth of LUAD cells.

**Conclusion:**

Our findings indicate that MDH1 may serve as a potential prognostic marker and a promising target for cancer immunotherapy.

## Introduction

Malignant neoplasms represent a major global health challenge, significantly affecting human health (1, 2). The continued societal impact of cancer necessitates further research into its causes and possible intervention targets. Oncology research typically focuses on clinical observations, genomic profiling, and histological features to explore the unique characteristics and heterogeneity of a specific cancer type. However, such research is restricted in its capacity to address the full complexity of cancer genetics and mechanisms. The advancements in artificial intelligence and bioinformatics has revolutionized cancer genomics, enabling large-scale analyses across multiple cancer types and facilitating a pan-cancer approach (3–7). This methodology integrates a wide range of oncogenic genomic data, helping to identify commonalities across different cancers and unravel common genetic polymorphisms and mechanisms of dysfunction (8). As a result, comprehensive tumor analysis provides new insights and approaches for the purpose of early clinical treatment and intervention of cancer.

Malate-aspartate shuttle (MAS) plays a crucial role in maintaining intracellular NAD(H) redox balance by transferring reducing equivalents across the mitochondrial membrane in pancreatic cancer (9). The NAD+/NADH pools in the cytosol and mitochondria function independently, and maintaining a balanced redox state within each compartment is essential for cellular proliferation and growth (10, 11). A continuous supply of cytosolic NAD+ is critical for glycolysis, which is supported by glyceraldehyde-3-phosphate dehydrogenase (10). The restoration of cytosolic NAD+ levels is achieved through the reversible conversion of oxaloacetate to L-malate, catalyzed by MDH1 (12) (**Supplementary Figure S1A**).

The tissue-specific expression of MDH1 reflects its role in meeting elevated aerobic metabolic demands. High MDH1 expression levels are found in cardiac, skeletal muscle, and brain tissues, while expression is lower in smooth muscle and kidney tissues, and minimal in liver tissue (13–15). MDH1 plays a critical enzymatic role in situations requiring increased glycolytic capacity to meet anabolic demands or to counteract NAD redox imbalance caused by mitochondrial dysfunction. The enzyme recycles glycolytic NADH, supporting the proliferation of cancer cells and activated lymphocytes (10), both of which are characterized by impaired mitochondrial function and an increased reliance on glycolysis.

Research indicates that MDH1 works with lactate dehydrogenase (LDH) to regenerate NAD+ during cellular proliferation. In cancer cells, depletion of MDH1 has been shown to slow down both proliferation rates and glucose uptake. In the context of Warburg metabolism, MDH1 is involved in the metabolic processes of proliferating cells, alongside LDHA, suggesting that therapeutic strategies targeting glycolysis in cancer cells should also consider inhibiting MDH1 (10). Additionally, suppression of MDH1 leads to mitochondrial dysfunction, which in turn affects ATP utilization for cytoskeletal dynamics (16). Moreover, increased expression of MDH1 in tumor cell-enriched regions by transcriptional digital spatial profiling, which were associated with poor prognosis of nasopharyngeal carcinoma (17). Furthermore, robust up-regulation of MDH1, coupled with conformational or post-translational modifications, has been consistently observed in prostate, breast and pancreatic ductal adenocarcinomas, highlighting its pivotal function in rewiring cancer cell metabolism (18, 19). Zhang et al. demonstrated that MDH1 protein expression was significantly elevated compared to corresponding normal cases via human tissue microarray analysis and that it was located in the cytosolic fraction. Additionally, MDH1 activity might lead to reduced cytosolic oxaloacetate levels, thereby promoting malate synthesis (20). Investigators have successfully synthesized compound 50, which functions as a potent inhibitor of both MDH1 and MDH2, thereby inhibiting the activity of the MAS in A549 lung cancer cells, as documented in the literature (21).

Malate dehydrogenase, an essential enzyme in both the MAS and the tricarboxylic acid (TCA) cycle, has been extensively studied for its metabolic significance. A decrease in MDH1 activity has been linked to cellular senescence (22). Conversely, a reduction in MDH1 levels has also been shown to trigger senescence (22). While the nuclear function of MDH1 has received less attention, it is known that under glucose-deprived conditions, MDH1 enhances the interaction between p53 and the promoters of its target genes (23). Additionally, a correlation has been established between MDH1 activity and the aggressiveness of certain cancers, with pancreatic cancer being a notable example (24).

Immunotherapy is widely regarded as a groundbreaking advancement in cancer treatment, representing the third major evolution in oncological therapies. Extensive research has demonstrated its therapeutic potential involved in diverse tumor types, containing lung, melanoma, and gastrointestinal cancers (25, 26). However, the clinical application of immunotherapy faces challenges, such as adverse side effects (27) and a relatively low response rate. These limitations have driven efforts to identify biomarkers that can predict treatment efficacy and refine therapeutic approaches to overcome resistance mechanisms. Most biomarker studies have relied on whole-exome sequencing or RNA sequencing (RNA-seq) of tumor samples, which provide a broad genetic overview and include biomarkers like programmed cell death-ligand 1 (PD-L1) (28), microsatellite instability (MSI) (29), and tumor mutation burden (TMB) (30). With the introduction of single-cell RNA sequencing (scRNA-seq), researchers can now study gene expression at the level of individual cells, offering a more detailed approach to identifying biomarkers that better predict immunotherapy responses.

Considering its essential metabolic roles and its reported involvement in various cancers, MDH1 may be an important player in a wide range of tumor types and tumorigenic processes. Nevertheless, a systematic, pan-cancer exploration into the specific roles of MDH1 is notably lacking in current scientific research. Studies focusing on a single cancer type may overlook the broader mechanistic significance of a target gene, as they fail to provide a complete perspective. Therefore, there is a critical need to examine the expression patterns of MDH1 across different cancer types, which is essential for guiding future experimental and clinical research.

**Figure 1:**
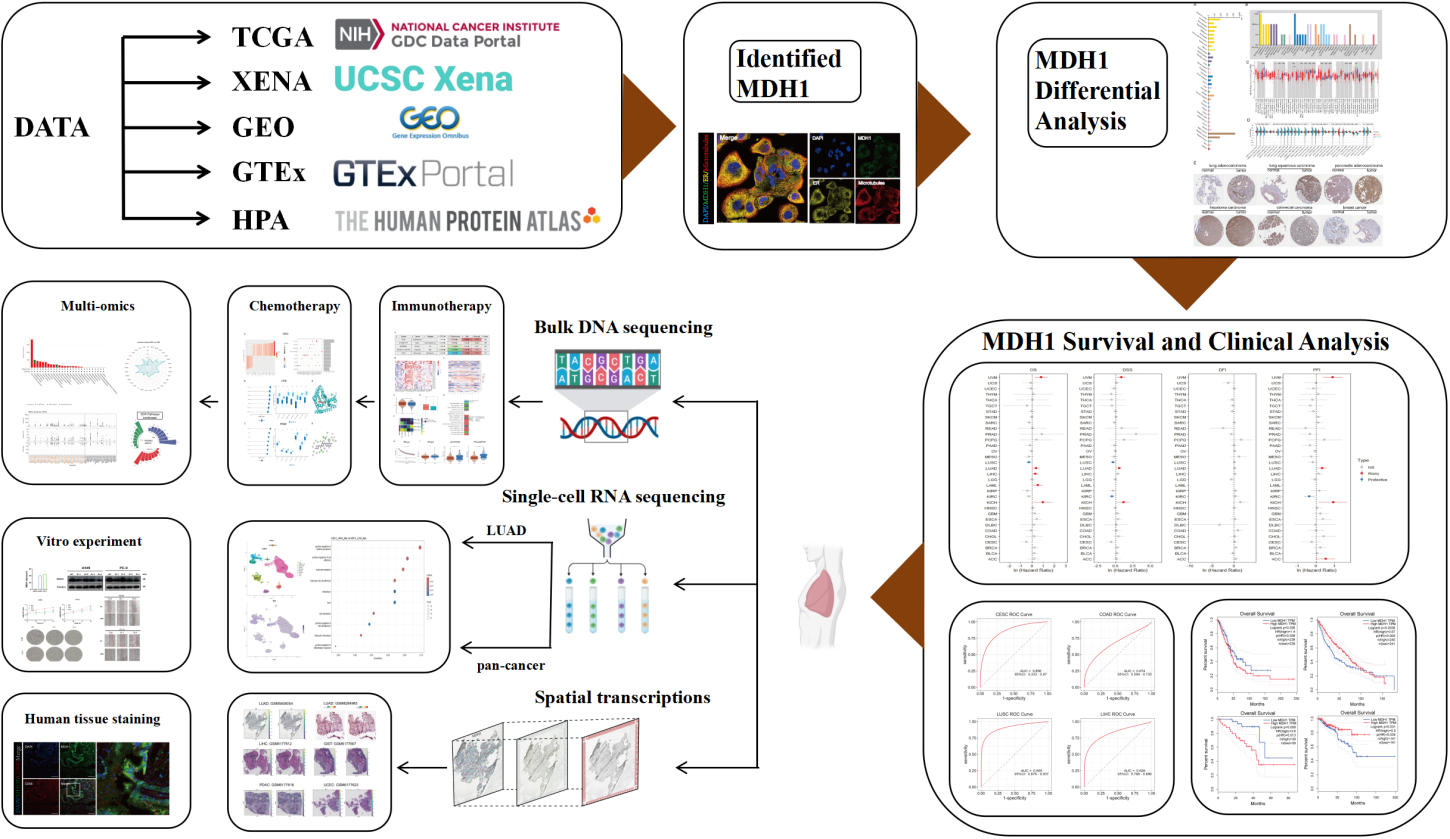
Schematic flowchart.

In this study, we have undertaken a comprehensive, multi-omics interrogation of MDH1 across a spectrum of malignancies, integrating paired neoplastic and adjacent-normal datasets. Our analyses have delineated robust associations between MDH1 abundance, clinical phenotypes, and inter-tumoral heterogeneity, with particular emphasis on its involvement in DNA-repair pathways, damage-response circuits, and immune-microenvironment crosstalk. Immunofluorescence validation has further established MDH1 as a quantitative indicator of macrophage infiltration. Complementary *in-vitro* experiments have corroborated its pro-tumorigenic impact in lung adenocarcinoma, while high-throughput drug profiling has nominated small-molecule activators of MDH1 for potential therapeutic exploitation. An overview of the analytical workflow is provided in **Figure 1**.

## Materials and methods

### MDH1 expression and localization data processing

In this investigation, the Human Protein Atlas (HPA) repository was used to obtain gene expression datasets and subcellular localization images for MDH1 across various tissues. Data on MSI and TMB were sourced from The Cancer Genome Atlas (TCGA). The conservation of the MDH1 gene across vertebrate species was visualized using the University of California, Santa Cruz (UCSC) Cancer Genomics Browser. The UCSC XENA platform was used to acquire transcriptome RNA-seq data and clinical profiles from TCGA for a wide range of cancer types, while the Genotype-Tissue Expression (GTEx) project provided data for normal tissue samples. RNA-seq data, quantified as transcripts per million (TPM), were extracted for both cancerous and adjacent (para-cancer) samples from TCGA. The ComBat algorithm, which is part of the “sva” package in R, serves as an effective tool to alleviate the impact of batch effects stemming from non - biological technical factors in gene expression data across multiple datasets during data preprocessing. To explore the mutational and copy number variation (CNV) landscapes of MDH1 in various cancer types, the cBioPortal for Cancer Genomics was utilized (31). Clinical data related to immune therapies were obtained from the Tumor Immunotherapy Gene Expression Resource (TIGER) (32). **Supplementary Table S1** summarizes the public data cohorts included in this study.

### Diagnostic and prognostic analysis

Recognizing the Cox proportional hazards model as a standard approach for survival analysis, this study applied it to a comprehensive cancer dataset sourced from the UCSC repository. The goal was to analyze the association between MDH1 expression and oncological outcomes. The diagnostic potential of MDH1 in cancer was assessed using Receiver Operating Characteristic (ROC) analysis. The Gene Expression Profiling Interactive Analysis 2 (GEPIA2) platform was used to examine correlations between MDH1 expression levels and key survival metrics, namely overall survival (OS) and disease-free survival (DFS). Additionally, survival data from multiple lung cancer cohorts were compiled from the Lung Cancer Explorer database (https://lce.biohpc.swmed.edu/lungcancer).

### Single-cell RNA sequencing

For this research, we accessed the GSE200972 dataset from the GEO repository, extracting 14 LUAD specimens for detailed analysis. We also integrated a diverse collection of single-cell datasets from various cancer types: GSE207422 (LUAD), GSE120575 [skin cutaneous melanoma (SKCM)], GSE145281 [bladder urothelial carcinoma (BLCA)], GSE169246 [triple-negative breast cancer (TNBC)], GSE212217 (UCEC), GSE229353 (lung carcinoma), and GSE235672 [glioblastoma multiforme(GBM)], all obtained from GEO. A subset of 54 samples from these datasets was selected for in-depth analysis. scRNA-seq analysis was performed using the “Seurat” R package. Cells were filtered based on the following criteria: expressed gene count per cell between 200 and 6000, with mitochondrial content less than 5%.

Batch effects were removed utilizing the harmony R package. Cluster-specific cell types were then identified using discrete cellular markers. Additionally, we obtained multiple scRNA-seq datasets from the Tumor Immunity Single Cell Center (TISCH) for supplementary analysis.

To assess cellular differentiation and progression, we used Monocle2 with its standard parameters to perform pseudotime trajectory analysis, organizing the cells along developmental pathways that were divided into distinct branches reflecting cellular progression or differentiation.

### Spatial transcriptome analysis

We randomly selected samples from each of the different cancer types across multiple GEO cohorts (GSE203612, GSE250636, GSE193460, GSE245704 and GSE194329) and Spatial Transcript Omics DataBase (https://db.cngb.org/stomics/) to create a spatial pan-cancer cohort. It consists of 9 different types of cancers, with at least three samples for each type of cancer. Spatial clustering was performed using a graph-based approach implemented in the Scanpy package (33). Principal component analysis (PCA)-based dimensionality reduction and subsequent spatial transcriptomic (ST) point grouping were executed via the RunPCA, FindNeighbors, and FindClusters algorithms. Leveraging histological data from hematoxylin and eosin (H&E)-stained tissue sections, clusters were annotated to clarify cell identities. Further analysis and visualization of the annotated clusters enabled the assessment of MDH1 and CD68 expression intensities as well as their spatial localization patterns within the tissue landscape.

### Immunogenic landscape assessment

Tumor Mutational Burden (TMB) was quantified by counting non-synonymous somatic mutations. Neoantigens from LUAD samples in TCGA were retrieved from The Cancer Immunome Atlas (TCIA) (34), and the tumor neoantigen burden (TNB) was calculated by aggregating the total predicted neoantigens.

### DNA damage response status assessment

DNA-damage-response (DDR) integrity was classified by scanning each tumor for non-silent variants within the seven core repair branches [Base Excision Repair (BER), Nucleotide Excision Repair (NER), Mismatch Repair (MMR), Homologous Recombination Repair (HRR), Non-Homologous End Joining (NHEJ), Fanconi Anemia (FA), or Translesion Synthesis (TLS)]; any lesion classified the sample as DDR-mutant (DDR-Mut), whereas absence of such events yielded DDR-wild-type (DDR-WT).

For MDH1, we integrated four orthogonal layers of genomic regulation. Somatic mutation and copy-number landscapes were retrieved from cBioPortal; promoter and gene-body methylation were profiled with UALCAN and refined by MEXPRESS (https://mexpress.ugent.be/). Clinically relevant alternative-splicing events were extracted via OncoSplicing (ClinicalAS tool) (35) and visualized with PanPlot, comparing PSI values between TCGA tumors and GTEx normal tissues for events spanning ≥3 cancer types.

### Analysis of drug sensitivity

The responsiveness of cancer cell lines (CCLs) to various drugs was evaluated using data from three sources: the PRISM (36), CTRP (37), and GDSC (38) databases. Both CTRP and PRISM assess drug sensitivity using the AUC, while GDSC uses the IC50. It is important to note that lower IC50 or AUC values indicate higher responsiveness to the treatment compounds.

### Molecular docking and molecular dynamics simulation

MDH1 structure was modelled with AlphaFold3 (39). Binding poses of candidate ligands from PubChem were predicted using AutoDockTools within an 80 × 80 × 80 Å grid centered at (65, 20, 180); complexes were inspected in PyMOL. To assess the stability of the BI-2536–MDH1 complex, 100-ns MDS was performed in GROMACS (40, 41).

### Cell culture

The LUAD cell lines, A549 and PC-9, normal bronchial epithelial cells (BEAS), and human monocyte cell line (THP-1) were obtained from the Shanghai Cell Bank, affiliated with the Chinese Academy of Sciences. These cells were cultured in growth medium containing 10% fetal bovine serum (FBS) and 1% penicillin-streptomycin mixture at 37°C. A549 and PC-9 cell lines were grown in DMEM, while THP-1 cells were cultured in RPMI 1640 medium.

### Sample collection

A total of thirty LUAD tissue specimens were obtained from the First Affiliated Hospital of Wenzhou Medical University. Participants provided their written consent to be involved in the study. BI-2536 was acquired from MedChemExpress (#HY50698, USA).

### Quantitative real time polymerase chain reaction analysis

RNA was isolated from both cell and tissue samples using Trizol reagent (Takara, Japan). Subsequently, cDNA synthesis was carried out with the PrimeScript RT enzyme kit. qRT-PCR was performed using the SYBR Green fluorescence detection system. Primer sequences utilized in this study are listed in **Supplementary Table S2**.

### Cell transfection

In this study, custom-designed MDH1 small interfering RNA (siRNA) and its respective scrambled non-targeting control (SiNC) were used for investigation. The specific sequences of the MDH1 siRNA are provided in **Supplementary Table S3**. LUAD cell lines A549 and PC-9 were transfected with the siRNA, and cells were collected 48 hours post-transfection for further analysis.

### Cell Counting Kit-8 assay

Following a 48-hour incubation, the transfected cells were harvested and plated into 96-well plates at a density of 5,000 cells per well. Cell proliferation was assessed every 24 hours using the CCK-8 assay, with absorbance measurements taken after a 1-hour incubation with the reagent. A549 and PC-9 cells were seeded into 96-well plates; 24 h later, they were exposed to a gradient of BI-2536 for an additional 24 h, after which viability was quantified with the CCK-8 assay.

### Transwell assay

Forty-eight hours post-transfection, 300 μl of serum-free medium containing 4 × 10^4 cells was added to the upper chamber, while the lower chamber was filled with 600 μl of medium supplemented with 20% FBS. After 24 hours, the medium was changed, and non-migratory cells were removed with cotton swabs. The migrated cells were then fixed and stained with 0.01% crystal violet. Finally, the cells that had migrated to different regions of the membrane were counted under a microscope.

### Wound healing assays

Following transfection, cells were seeded in a 6-well plate and cultured until they reached 80%–90% confluence. A linear wound was then created by carefully scraping the cell monolayer using a 1000 μl pipette tip. Detached cells were removed by rinsing with phosphate-buffered saline (PBS). Wound closure was tracked at designated time points by capturing images using an inverted microscope.

### Lactate measurement

After transfection, the cells were cultured in fresh medium for an additional 48 hours. The lactate concentration in the culture supernatant was subsequently measured using spectrophotometric measurements.

### M2 macrophage related transwell assay and infiltration assay

For the invasion assay, Matrigel was diluted at a ratio of 1:10 and applied at 70 μL per well to the upper chamber of the transwell system. In the migration assay, 600 μL of medium containing 20% FBS was added to the lower chamber, while 9 × 10^4 cells in 300 μL of serum-free medium were seeded in the upper chamber. For the M2 macrophage infiltration assay, THP-1 cells were differentiated into macrophages by treating with 100 ng/mL of phorbol 12-myristate 13-acetate (PMA; MCE, USA) for 24 hours. Polarization towards the M2 phenotype was induced by incubating the cells with 20 ng/mL of interleukin 4 (IL-4; MCE, USA) for 48 hours. The M2 macrophages were then cultured in the upper chamber with serum-free medium, while A549 and PC-9 cells were cultured in the lower chamber. After 48 hours of incubation, cells were fixed for further analysis.

### Immunofluorescence assay

A total of thirty LUAD tissue sections, embedded in paraffin, were prepared for IF staining. The sections were deparaffinized, treated with 3% hydrogen peroxide to block endogenous peroxidase activity, and underwent antigen retrieval. They were then incubated with 2% bovine serum albumin for 30 minutes to prevent non-specific binding. After blocking, the sections were incubated with primary antibodies against MDH1 (rabbit polyclonal, 1:100 dilution, Proteintech) and CD68 (mouse monoclonal, 1:3000 dilution, Proteintech). Following this, secondary antibodies were applied: CoraLite488-labeled Goat Anti-Mouse IgG and CoraLite594-labeled Goat Anti-Rabbit IgG. Finally, cell nuclei were visualized by counterstaining with 4’,6-diamidino-2-phenylindole (DAPI).

### Western blot

Cell samples were collected 48 hours after transfection, and WB analysis was performed according to established protocols (42). Total protein was extracted using RIPA buffer, supplemented with phosphatase and protease inhibitors. The proteins were then denatured and prepared for WB analysis. Primary antibodies targeting MDH1 (1:5000 dilution, Proteintech), HK2(1:5000 dilution, Proteintech), LDHA (1:2000 dilution, Proteintech) and Tubulin (1:10000 dilution, Proteintech) were used in this experiment.

### Elisa

Medium was collected in chilled sterile tubes, cleared by 4°C centrifugation (1000 × g, 10 min), and aliquoted into microtubes. Cytokine quantification (IL-10 and TNF-α) was performed with the commercial ELISA reagents (Solarbio, Beijing) according to the vendor’s specifications.

### Cellular thermal shift assay

CETSA was carried out following a published protocol (43) with minor modifications. Briefly, A549 cell were exposed to BI-2536 (500 nM) or vehicle for 24 h, harvested, and resuspended in PBS supplemented with protease inhibitors. The suspension was split into seven aliquots that were individually heated (55, 60, 65, 70, 75, 80, 85 °C) for 3 min. Thermal‐treated samples were snap-frozen in liquid nitrogen for 8 h, thawed at 26°C for 6 min, and the freeze–thaw cycle was repeated twice to ensure complete lysis. After centrifugal clarification, MDH1 levels in the supernatants were determined by immunoblotting.

### Statistical analysis

The mean plus the standard error of the mean was shown after performing at least three independent experiments for data analysis. Statistical analyses were carried out using R version 4.1.1 and GraphPad Prism 10.0. Prior to statistical testing, data were assessed for normality using the Shapiro-Wilk test and for homogeneity of variances using Levene’s test. For group comparisons, if data met the assumptions of normality and equal variances, one-way ANOVA with Tukey’s *post-hoc* correction for multiple comparisons was used. If assumptions were violated, the non-parametric Kruskal-Wallis test with Dunn’s *post-hoc* correction was applied instead of Student’s t-tests. For correlations between variables, Pearson’s correlation coefficient was used for parametric data, and Spearman’s rank correlation coefficient was used for non-parametric data. To control for multiple hypothesis testing, the Benjamini-Hochberg procedure was applied to adjust p-values, and a corrected p-value threshold of less than 0.05 was considered statistically significant.

## Results

### MDH1 expression in multiple cancers

**Figure 2:**
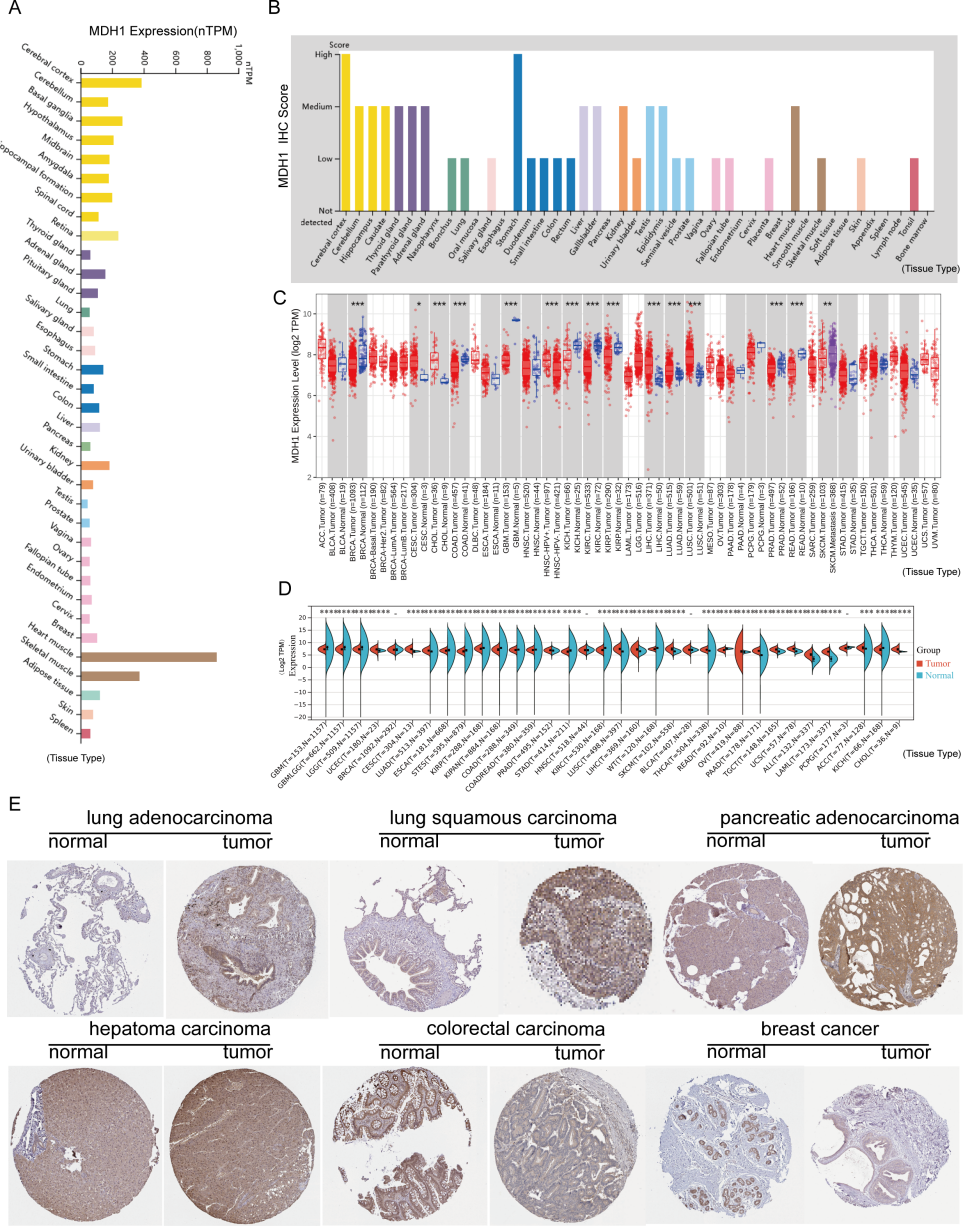
MDH1 expression profiles. MDH1 mRNA **(A)** and protein expression **(B)** levels generated by GTEx, HPA. MDH1 expression explored by TCGA dataset **(C)** and TCGA-GTEx dataset **(D)**. Immunohistochemistry staining of MDH1 in HPA **(E)**. *P < 0.05, **P < 0.01, ***P < 0.001.

We analyzed MDH1 gene expression in a cohort of healthy subjects using data from the GTEx project. Our results showed that MDH1 was widely transcribed across various organs, with particularly high expression levels observed in the cerebral cortex and basal ganglia, as shown in **Figure 2A**. Additionally, we examined MDH1 protein expression in a diverse group of healthy individuals, using data from the HPA. This analysis identified a significant upregulation of MDH1 in the cerebral cortex and stomach, as illustrated in **Figure 2B**.

In the subsequent phase of our study, we explored MDH1 mRNA expression in cancerous tissues involved in 33 cancer types, utilizing data from TCGA. Our results identified significant upregulation of MDH1 mRNA in several cancers, such as cholangiocarcinoma (CHOL), cervical squamous cell carcinoma and endocervical adenocarcinoma (CESC), hepatocellular carcinoma (LIHC), head and neck squamous cell carcinoma (HNSC), LUAD, lung squamous cell carcinoma (LUSC), uterine corpus endometrial carcinoma (UCEC) and stomach adenocarcinoma (STAD). Conversely, MDH1 mRNA expression was found to be reduced in breast invasive carcinoma (BRCA), colon adenocarcinoma (COAD), glioblastoma multiforme (GBM), kidney chromophobe (KICH), kidney renal clear cell carcinoma (KIRC), and thyroid carcinoma (THCA), as illustrated in **Figure 2C**.

Additionally, by integrating data from GTEx and TCGA, we observed significant overexpression of MDH1 in 19 out of the 33 cancer types analyzed (**Figure 2D**). We further examined IHC results for MDH1 in the HPA database, which showed strong expression levels in LUAD, LUSC, PAAD, and LIHC, while expression was lower in COAD and BRCA. Collectively, these results underscore the significant overexpression of MDH1 in various cancer types, highlighting its essential involvement in tumorigenesis, as depicted in **Figure 2E**.

### MDH1 is associated with clinicopathological traits

**Figure 3:**
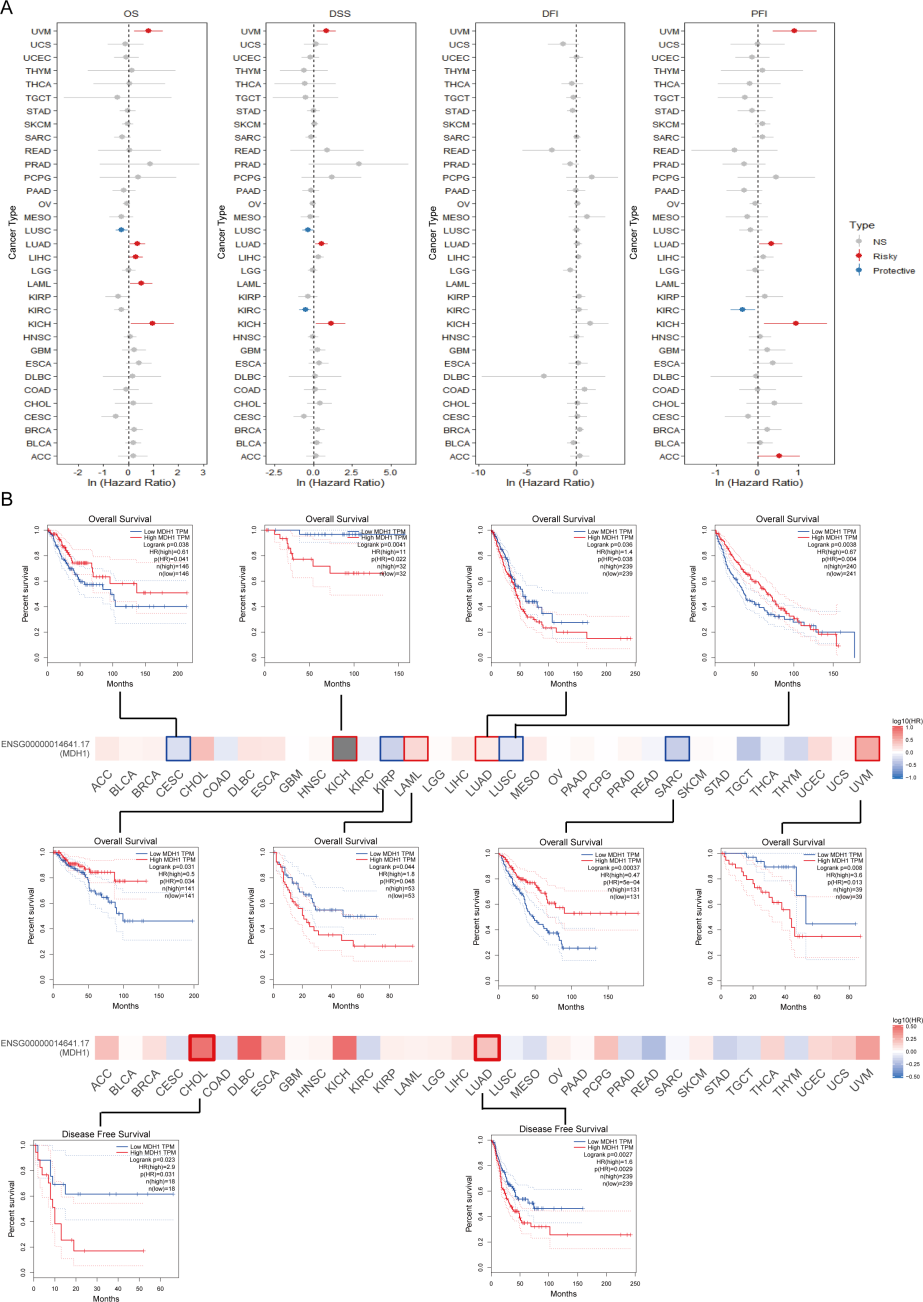
Survival analysis of MDH1 in pan-cancer. Univariable cox regression analysis for OS, DSS, DFI and PFI **(A)**. The survival maps and curves **(B)**.

**Figure 4:**
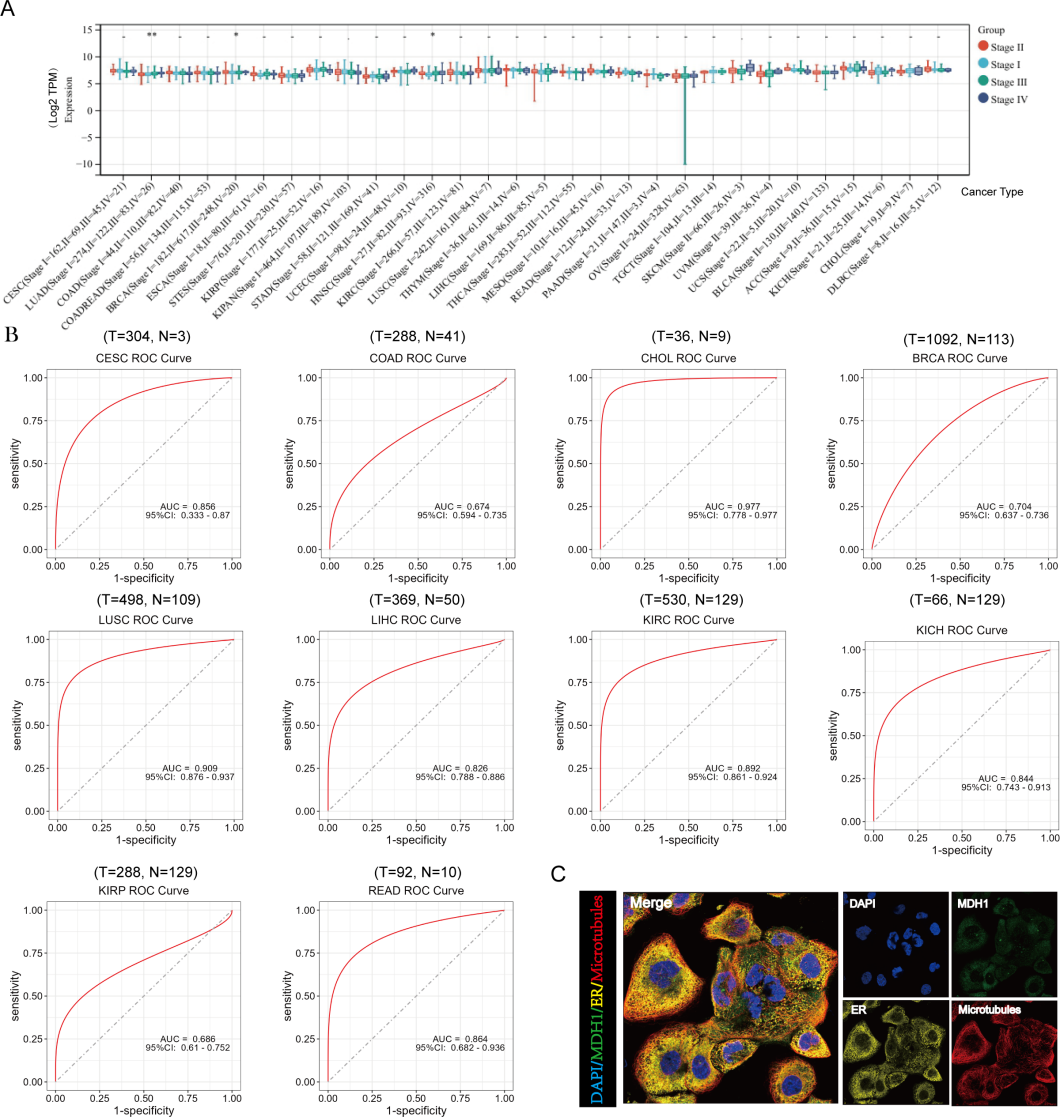
Analysis of clinical features of MDH1 in pan-cancer. The association between MDH1 expression and stage **(A)**. The ROC curves of MDH1 in CESC, COAD, CHOL, BRCA, LUSC, LIHC, KIRC, KICH, KIRP and READ **(B)**. Subcellular localization of MDH1 **(C)**.

To explore the clinical implications of MDH1 expression, we conducted a comprehensive analysis to assess its relationship with patient survival metrics, including OS, DSS, DFS, and PFS, across 33 cancer types in the TCGA database. Our result unraveled that MDH1 expression was an adverse factor for OS, DSS, and PFI in LUAD, Uveal Melanoma (UVM), and KICH (**Figure 3A**). Furthermore, Kaplan-Meier survival analysis showed that MDH1 expression significantly impacted OS. Specifically, it acted as a risk factor for OS in LUAD, KICH, acute myeloid leukemia (LAML), and UVM, while it was associated with improved OS in cervical squamous cell carcinoma (CESC), KIRP, LUSC, and sarcoma (SARC), as shown in **Figure 3B** (**Supplementary Table S4**). Additionally, analysis of TCGA data identified a positive correlation between MDH1 expression and tumor stage, as well as the metastatic (M) and nodal (N) classifications in LUAD (**Figure 4A**, **Supplementary Figures S2A, B**). These findings indicate that MDH1 expression is most strongly associated with the aggressiveness of LUAD. To further validate these results, we extracted data from 40 LUAD patient cohorts in the TIGER database and Lung Cancer Explorer (https://lce.biohpc.swmed.edu/lungcancer/). Survival analysis in these cohorts showed that high MDH1 expression was linked to poorer survival outcomes in most LUAD patient groups, as detailed in **Supplementary Figures S2C-E**.

Additionally, the diagnostic significance of MDH1 across various tumors was assessed utilizing ROC curves. The results identified that MDH1 exhibited high diagnostic accuracy for CESC, CHOL, BRCA, LUSC, LIHC, KIRC, KICH, and READ, as indicated by the area under the curve (AUC) presented in **Figure 4B**. Furthermore, MDH1 protein expression patterns were analyzed using the HPA database, revealing that MDH1 was mainly localized in the cytoplasm and co-localized with the endoplasmic reticulum. These findings indicate that MDH1 may be involved in lipid and protein biosynthesis and maturation, as shown in **Figure 4C**.

### Investigations into genomic variations and instability pertaining to MDH1

Genomic techniques provide a solid framework for understanding oncogenic processes (44). MDH1 gene amplification was most commonly observed in non-small cell lung carcinoma (NSCLC), UCEC, BLCA, and ovarian cancers, while mutations were predominantly found in COAD. Additionally, a significant frequency of SNVs was detected in SKCM, UCEC, and KICH (**Supplementary Figure S3A**). A detailed analysis of the mutational landscape of MDH1 is presented in **Supplementary Figure S3B**. On the epigenetic level, the methylation status of various sites within the MDH1 promoter and gene body showed a strong positive correlation with mRNA expression levels in most tumor samples (**Supplementary Figure S3C**).

Through computational analysis, we successfully identified the m6A modification sites within the MDH1 gene sequence, as shown in **Supplementary Figure S3D**. Our analysis revealed four m6A sites with a high degree of confidence, located at nucleotide positions 2287, 2350, 6534, and 16326. Dysregulated gene expression in cancer is often linked to abnormal DNA methylation patterns, as highlighted by previous studies (45). To explore the potential connection between aberrant transcriptional profiles of MDH1 and its methylation status, we utilized the UALCAN database (46) to examine methylation patterns in both cancerous and non-cancerous tissues. By comparing the differential methylation between cancerous and non-cancerous samples in the UALCAN database (**Supplementary Figure S4A**), we observed a correlation between higher MDH1 expression levels in LUAD, BLCA, thyroid carcinoma (THCA), and testicular germ cell tumors (TGCT) and reduced methylation levels (**Supplementary Figure S4A**). Furthermore, in the MEXPRESS database, methylation changes at sites cg14736172 (R = -0.098) and cg10164987 (R = 0.107) in LUAD showed an inverse correlation with MDH1 expression levels (**Supplementary Figure S3E**). Additionally, changes in methylation at sites cg02914501 (R = -0.093), cg17972846 (R = -0.136), cg24055349 (R = -0.188), cg09303977 (R = -0.124), cg19484848 (R = 0.094), and cg14458626 (R = 0.092) in SKCM were found to correlate with MDH1 expression (**Supplementary Figure S3F**).

A pie chart representation of CNVs revealed the widespread occurrence of MDH1 CNVs across all 33 cancer categories in the TCGA dataset, as shown in **Supplementary Figure S3G**. Moreover, an increased proportion of tumors exhibited copy number gains for the MDH1 gene, while copy number deletions were observed in only a small fraction of the samples (**Supplementary Figure S3H**). A higher incidence of MDH1 CNVs was particularly noted in BRCA, LUSC, BLCA, ESCA, LUAD, and CESC (**Supplementary Figure S3I**).

### Alternative MDH1 splicing is related with clinical prognosis

Distinct alterations in gene splicing patterns within tumor cells can drive the progression of malignancy. Detecting alternative splicing (AS) events may therefore enhance prognostic and diagnostic assessments for patients, as suggested by previous studies (47). A pan-cancer analysis showing the percent spliced-in (PSI) values for the MDH1_alt_3prime_143356 event is presented in **Supplementary Figure S5A**. Compared to normal tissue samples, elevated PSI levels were observed in kidney renal KIRP, LIHC, LUSC, PCPG, and SKCM, whereas a contrasting pattern was noted in KICH and THYM. **Supplementary Figure S5B** summarizes the statistical differences in PSI between tumor and adjacent normal tissues, along with their prognostic significance. Kaplan-Meier survival curves revealed a relationship between PSI of MDH1 AS events and survival outcomes in select cancers, as shown in **Supplementary Figure S5C**. Overall, these findings suggest that AS events in MDH1 may take a significant role in the progression of various cancers.

### Variations in mutational profiles were discerned across distinct MDH1 subgroups

As shown in **Supplementary Figure S6A**, both TMB and TNB scores exhibited a progressive and significant increase with higher MDH1 expression, suggesting a potential association between MDH1 and enhanced immunogenicity as well as tumor heterogeneity in LUAD. Additionally, MDH1 showed strong positive associations with both synonymous (r = 0.19, p < 0.001) and non-synonymous (r = 0.19, p < 0.001) mutations in LUAD samples, as illustrated in **Supplementary Figure S6C**. Using the OncodriveCLUST algorithm, PPP3CA was identified as a shared driver gene in both the MDH1-L and MDH1-M cohorts, while no driver gene was detected in the MDH1-H group, as presented in **Supplementary Figure S6B**. A bar plot demonstrated a decrease in PPP3CA mutation frequency with increasing MDH1 expression, as shown in **Supplementary Figure S6D**. Significantly mutated genes were identified in the MDH1-L+M groups, visualized in the forest plot (**Supplementary Figure S6E**). In the MDH1-L+M group, the most frequently mutated genes were ACACB, PTPRC, and ZNF676. A ternary plot was used to depict the distribution of all non-silent mutations across the three MDH1 groups, including the 10 most common non-silent mutations (TP53, AMT, PRKDC, FANCM, BRCA2, POLE, HFM1, MSH4, ATR, and BRIP1; **Supplementary Figure S7A**). It was found that the top 10 mutated genes were more likely to occur in the MDH1-L group (**Supplementary Figure S6F**). Additionally, a higher frequency of co-occurring and mutually exclusive mutations was observed with decreasing MDH1 levels, indicating that MDH1 is associated with increased somatic mutation activity in LUAD (**Supplementary Figure S6G**). DNA damage response (DDR) genes are frequently mutated in LUAD, and these alterations are associated with favorable immune checkpoint inhibitors (ICIs) outcomes in LUAD patients, as reported in the literature (48). The mutation rates of eight DDR pathways (CPF, HRR, NER, FA, MMR, BER, NHEJ, and TLS) across different MDH1 groups are summarized in **Supplementary Figure S6H**.

The integrity of the cancer genome critically depends on the efficacy of DNA repair processes, including DNA methyltransferase (DNMT), MMR, and HRR. Moreover, the heatmap revealed a positive correlation between MDH1 expression and DNMT, MMR, and HRR-related genes in the majority of tumors (**Supplementary Figures S6I**, **S7B**).

### MDH1 is associated with immune function and participates in multiple oncogenic pathways

To explore the functions of MDH1 in the immune microenvironment, we analyzed TMB and MSI. As shown in **Supplementary Figure S8A**, a positive correlation was observed between MDH1 expression levels and TMB across various cancer types, including BLCA, BRCA, COAD, DLBC, HNSC, LUAD, READ, SKCM, STAD, and UCEC. Additionally, MDH1 expression was positively associated with MSI in COAD, STAD, and UCEC, whereas it showed an adverse relationship with MSI in LUAD and PRAD, as illustrated in **Supplementary Figure S8C**.

Next, we investigated the correlation between MDH1 expression and various immune cells. We revealed that MDH1 expression was positively association with both M2 and M1 macrophages. In contrast, MDH1 expression was negatively associated with regulatory T cells, memory B cells, and plasma cells (**Supplementary Figure S8B**).

Data from Spatial DB, obtained through spatial transcriptomic analysis, unraveled significant overlap in the expression profiles of MDH1 and the macrophage marker CD68, suggesting potential co-localization of these genes (**Supplementary Figure S8D**). Furthermore, single-cell RNA sequencing data from TISCH and HPA confirmed the presence of MDH1 transcripts in both macrophages and malignant tumor cells across most of the cancer types studied (**Supplementary Figures S8E, F**).

To elucidate the immunological divergence under MDH1-based stratification, we next examined how intrinsic immune phenotypes aligned with MDH1 abundance. Tumors falling into the MDH1-low set were strongly skewed toward a C3 (inflammation-centric) milieu, whereas those with elevated MDH1 expression were preferentially classified as C1 (wound-healing) or C2 (IFN-γ–polarized) immune subtypes (**Supplementary Figure S9A**).

### LUAD scRNA seq analysis

**Figure 5:**
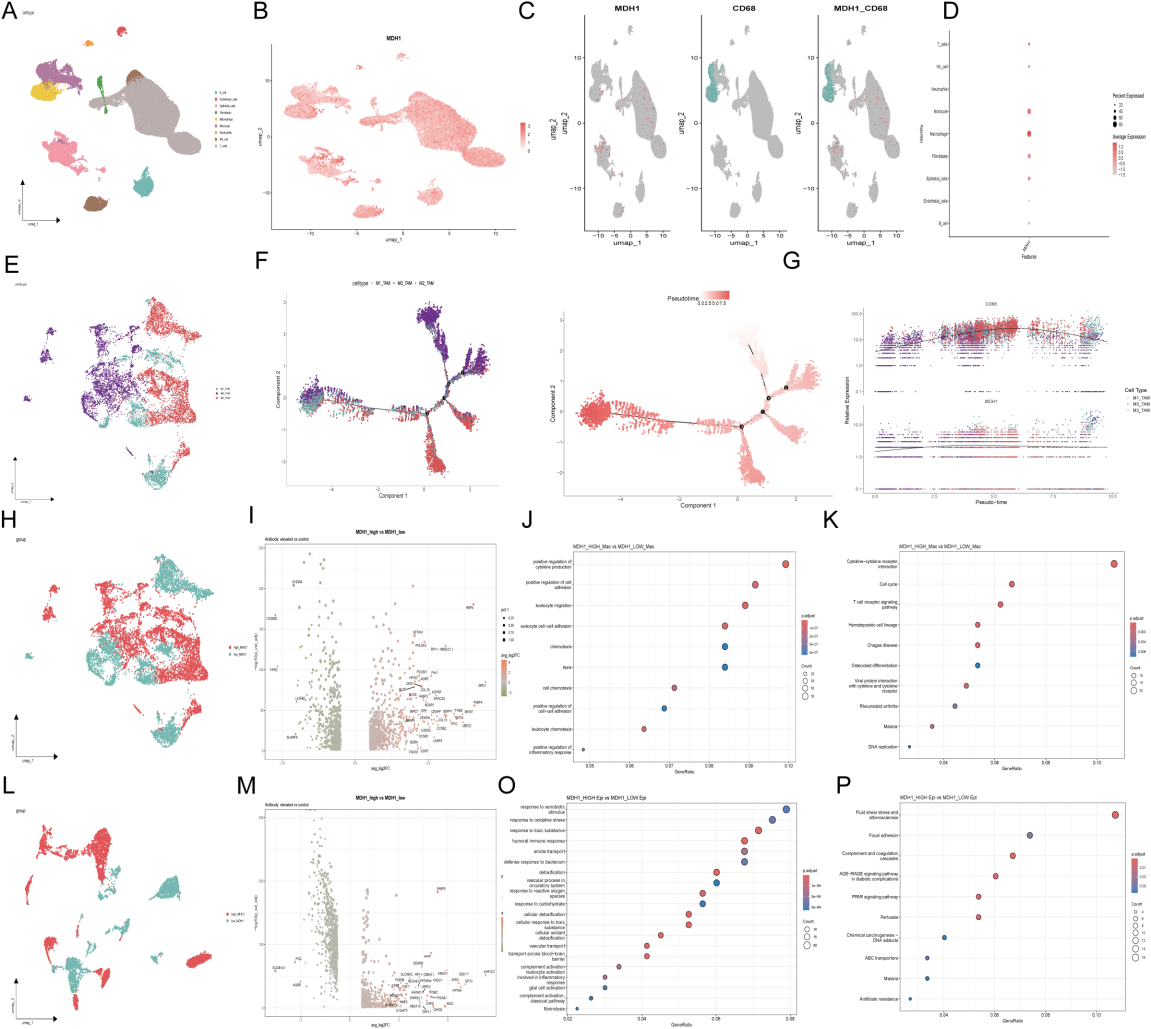
The exploration of MDH1 in scRNA dataset GSE200972. Umap diagram of 9 celltypes **(A)**; umap distribution diagram of MDH1 expression **(B)**; umap distribution diagram of MDH1 and CD68 **(C)**; Correlation with MDH1 and celltypes **(D)**;Umap diagram of macrophage subgroup **(E)**. Umap diagram of 9 celltypes **(A)**; umap distribution diagram of MDH1 expression **(B)**; umap distribution diagram of MDH1 and CD68 **(C)**; Correlation with MDH1 and celltypes **(D)**; Umap diagram of macrophage subgroup **(E)**. Trajectory reconstruction of all macrophage cells in TNBC, with a color code for cell types, pseudo-time **(F)**. The branched heatmap indicates the dynamics of the expression of MDH1 and CD68 during macrophage cells transdifferentiation **(G)**. In macrophage subgroup, umap diagram of MDH1-high and MDH1-low groups **(H)**, Volcanic map of differential genes **(I)**, GO **(J)** and KEGG **(K)**; In epithelial cell subgroup, umap diagram of MDH1-high and MDH1-low groups **(L)**,Volcanic map of differential genes **(M)**, GO **(O)** and KEGG **(P)**.

To further investigate the function of MDH1 in LUAD and validate the previously obtained analytical results, we utilized scRNA-seq data from the GSE200972 repository, which includes gene expression profiles of 93,201 cells derived from 14 LUAD samples. We have implemented rigorous quality control procedures (**Supplementary Figure S10A**). Dimensionality reduction of the gene expression data was performed using principal component analysis (PCA), revealing 33 distinct cell clusters. The cellular identity of each cluster was then annotated based on immunological markers described in the literature (49, 50) (**Figure 5A**). As expected, MDH1 was predominantly expressed in macrophages and epithelial cells (**Figures 5B, D**). Moreover, the expression profile of MDH1 showed a significant correlation with the macrophage-specific marker CD68 (**Figure 5C**).

To further explore the relationship between MDH1 and the dynamic evolution of macrophages in LUAD, we purified monocyte- and macrophage-enriched subpopulations and assigned them to M1, M0, or M2 phenotypes using marker genes previously established in the literature (51, 52) (**Figure 5E**). Using the Monocle2 toolkit, we ordered single cells along a pseudo-temporal developmental trajectory, which revealed a distinct and uniform maturation pathway for macrophages (**Figure 5F**). Notably, the expression patterns of MDH1 and CD68 closely matched the macrophage’s dynamic evolutionary trajectory (**Figure 5G**).

Following this, we categorized macrophages into high-MDH1 and low-MDH1 groups based on their MDH1 expression levels. A differential gene expression analysis was then conducted, with a log2 fold change > 0.5 and a p-value < 0.05 as the cutoff criteria (**Figures 5H, I**). In the biological process (BP) category, the differentially expressed genes (DEGs) were enriched in the positive regulation of cytokine production, cell adhesion, and leukocyte-related functions. KEGG pathway analysis revealed that these DEGs were predominantly involved in cytokine-cytokine receptor interactions, cell cycle regulation, and T cell receptor signaling, among other pathways (p< 0.05) (**Figures 5J, K**).

Given the potential role of MDH1 in epithelial cells, we also stratified epithelial cells into high-MDH1 and low-MDH1 groups, followed by differential gene expression analysis using the same criteria (**Figures 5L, M**). The DEGs in this category were associated with cellular migration, apoptosis, and oncogenic processes. KEGG pathway analysis indicated that these DEGs were primarily involved in oxidative stress responses and immune reactions (**Figures 5O, P**).

### The predictive value of MDH1 in immunotherapy cohort

**Figure 6:**
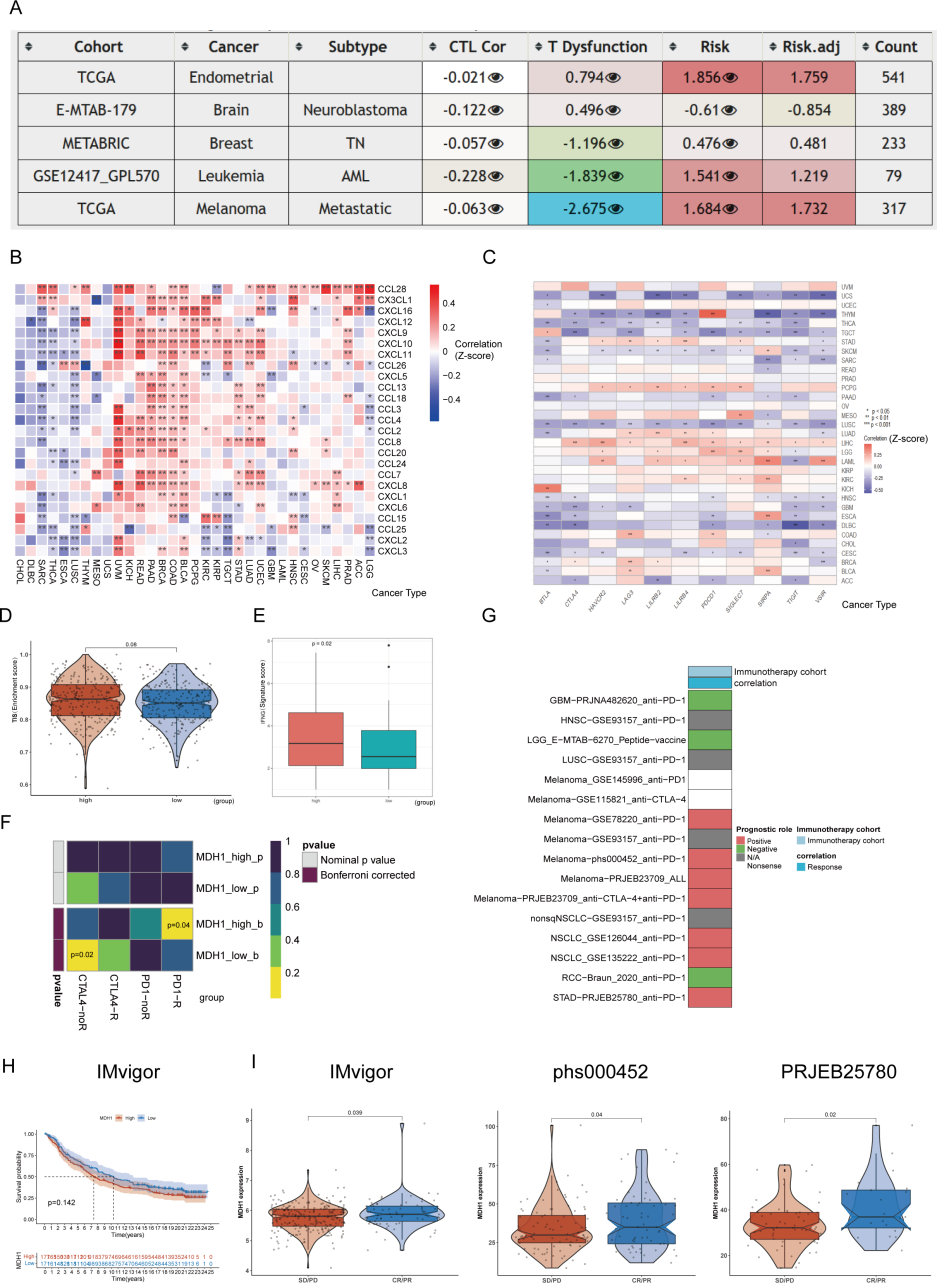
The prognosis value of MDH1 in immunotherapy. The table shows the correlations between MDH1 expression and CTL, CTL dysfunction, and risks **(A)**. The heatmap shows the correlations between MDH1 and chemokines **(B)**, immune checkpoint **(C)**. Comparing the difference between MDH1 and TIS scores **(D)**, IFG index **(E)**. Prediction of the response to anti-PD1 and anti-CTLA4 inhibitors in two MDH1 expression groups by the submap algorithm **(F)**. The correlation between MDH1 and response in immunotherapy **(G)**. K–M curves based on MDH1 expression in IMvigor cohort **(H)**. Comparing the MDH1 expression in IMvigor, phs000452 and PRJEB25780 cohorts **(I)**.

Initially, the influence of MDH1 on immune cell infiltration was assessed. The results revealed a positive correlation between MDH1 expression and the infiltration of myeloid-derived suppressor cells (MDSCs) across various tumor types, as illustrated in **Supplementary Figure S11A**. Next, cytotoxic T lymphocytes (CTLs), which are an essential functional subset of CD8+ T cells, were examined. Impaired CTL function is a critical factor in tumor immune evasion and resistance to immunotherapy, as highlighted in the literature (53). As depicted in **Figure 6A**, MDH1 expression showed an inverse correlation with CTL activity in UCEC, metastatic melanoma, neuroblastoma, leukemia, and TNBC.

Furthermore, the correlation between MDH1 and immunomodulatory genes was assessed. MDH1 showed a significant positive association with most chemokines and chemokine receptors in UCEC, LUAD, STAD, TGCT, KIRP, KIRC, PCPG, BLCA, COAD, BRCA, PAAD, READ, KICH, and UVM (**Figures 6B, C**). Additionally, we found that MDH1 expression was positively correlated with immune checkpoint genes in UCEC, STAD, PCPG, LUAD, LIHC, LGG, LAML, KIRC, BRCA, and BLCA.

Next, we explored the predictive value of MDH1 for immunotherapy response in the TCGA-LUAD cohort. As expected, the MDH1-high group exhibited higher TIS scores and IFNG expression compared to the MDH1-low group (**Figures 6D, E**). Moreover, we evaluated the sensitivity to anti-PD1 and anti-CTLA4 immunotherapy utilizing the SubMap algorithm. As anticipated, higher MDH1 expression in LUAD patients increases the likelihood of benefiting from immunotherapy (**Figure 6F**).

To validate our hypothesis, we analyzed 16 immunotherapy cohorts from TIGER. As shown in **Figures 6G-I**, elevated MDH1 expression was associated with better sensitivity to immunotherapy in the majority of the cohorts.

### Pan-cancer immunotherapy scRNA seq analysis

**Figure 7:**
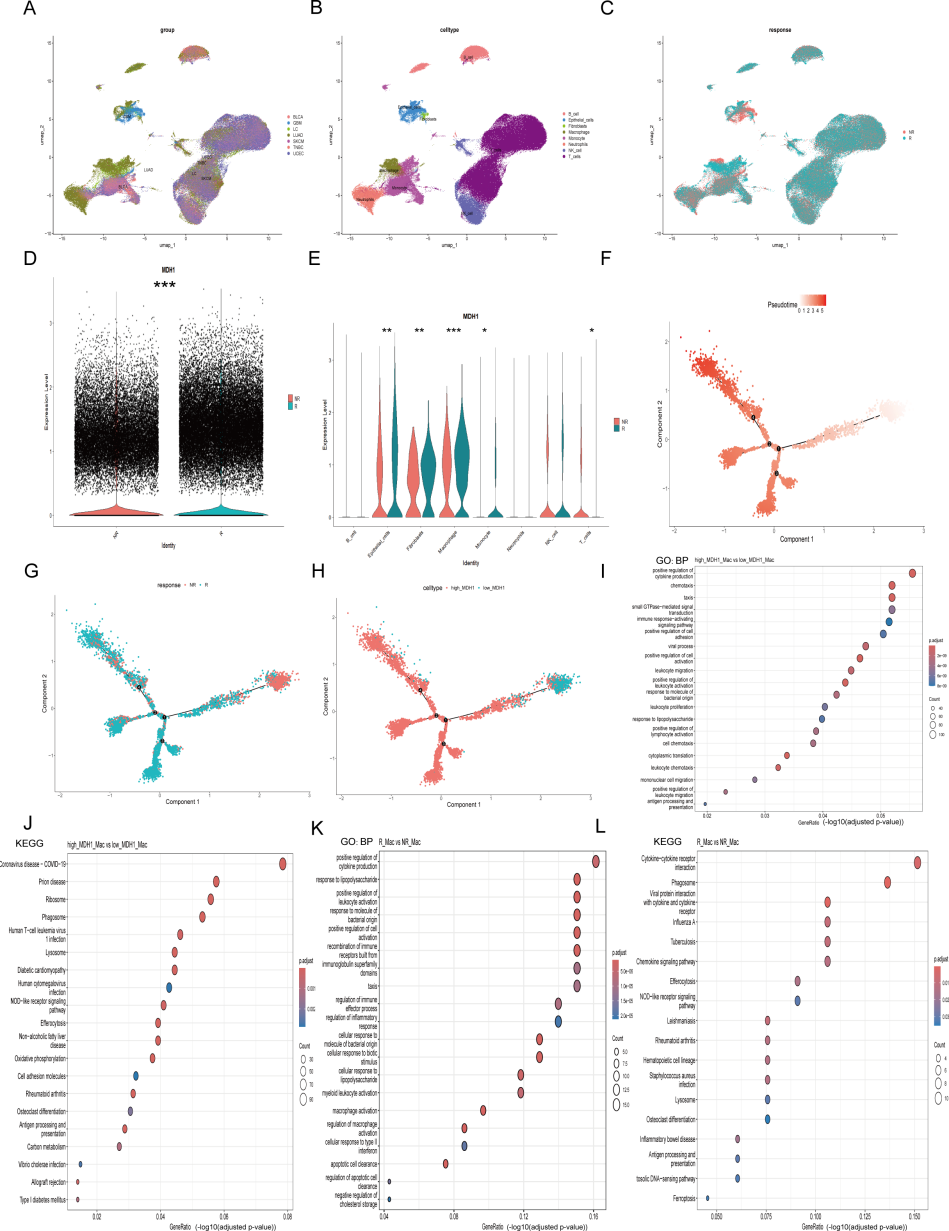
The exploration of MDH1 in pan-cacer scRNA dataset. Umap diagram of cancer cohorts **(A)**, celltypes **(B)**, immunotherapy responsiveness **(C)**. The proportion chart of celltypes **(D)** and immunotherapy responsiveness **(E)** in each cancer cohort. MDH1 expression difference in immunotherapy responsiveness **(F)** and celltypes **(G)**. The correlation MDH1 and celltypes **(H)**. In macrophage subgroup, the GO and KEGG analysis in MDH1-high and -low group **(I)**, the GO and KEGG analysis in R and NR group (J). *P < 0.05, **P < 0.01, ***P < 0.001.

A total of seven scRNA-seq datasets were utilized to explore the association between MDH1 expression levels and responses to immunotherapy, with the highest representation from UCEC samples (n = 17), LUAD (n = 11), and BLCA (n = 10) (**Supplementary Table S5**). Firstly, we have implemented rigorous quality control procedures (**Supplementary Figure S10B**). To identify unique cellular populations, we applied uniform manifold approximation and projection (UMAP) for dimensionality reduction and clustering of cells from the seven scRNA-seq cohorts, which included patients treated with ICIs across seven different cancer types (**Figures 7A, B**, **Supplementary Figure S12A**). **Figure 7C** shows the UMAP distribution of immunotherapy responsiveness. Among these cells, LUAD (GSE207422) had the largest proportion of macrophages, while GBM (GSE235672) had the largest proportion of epithelial cells (**Supplementary Figure S13A**).

Consistent with our previous hypothesis, the group that was sensitive to immunotherapy exhibited elevated MDH1 expression levels (**Figure 7D**). However, the distribution of cellular composition between the two groups was found to be nearly identical (**Supplementary Figure S13B**). We then conducted a comparative analysis of MDH1 expression levels across various cell types in the two cohorts. Interestingly, we observed a notable increase in MDH1 expression in the immunotherapy-sensitive set across nearly all cell types, which was an unexpected finding (**Figure 7E**). Furthermore, as previously noted, MDH1 expression remained strongly correlated with macrophages and epithelial cells (**Supplementary Figure S13C**).

**Figure 7F** shows the stratification of macrophages into distinct clusters, with clear separation observed between patients who responded to immunotherapy (R) and those who did not (NR). This highlights the presence of macrophage subset-specific features that may influence responses to immunotherapeutic interventions. Trajectory analysis revealed that the differentiation trajectories of macrophages in the high MDH1 group were similar to those in the R group (**Figure 7G, H**).

Next, we extracted the macrophage subgroup (**Supplementary Figure S13D**) and performed differential gene analysis between responders and non-responders, as well as between the MDH1-high and MDH1-low groups. The DEGs in the BP category were enriched in the positive regulation of cytokine production, cell adhesion, and leukocyte-related functions (**Figure 7I**). KEGG pathway analysis indicated that these DEGs were primarily enriched in the T cell receptor signaling pathway, NOD-like receptor signaling pathway, and antigen processing and presentation (**Figure 7J**). The KEGG and GO analyses of the DEGs between the R and NR groups showed trends that were largely consistent with the previous findings (**Figures 7K, L**).

Furthermore, we extracted the epithelial cell subgroup (**Supplementary Figure S13E**) and conducted differential gene analysis between the R and NR groups, as well as between the MDH1-high and MDH1-low groups. The DEGs in the BP category were involved in cell migration and epidermis development (**Supplementary Figure S13F**). KEGG analysis revealed that these DEGs were mainly involved in apoptosis and carcinogenic pathways (**Supplementary Figure S13G**). KEGG and GO analyses of the DEGs between the R and NR groups exhibited trends that were largely consistent with the previous outcomes (**Supplementary Figures S13H, I**).

In summary, the data strongly suggest a temporal and spatial concordance between cells with high MDH1 expression and those that respond to immunotherapy.

### Drug sensitivity analysis in LUAD

**Figure 8:**
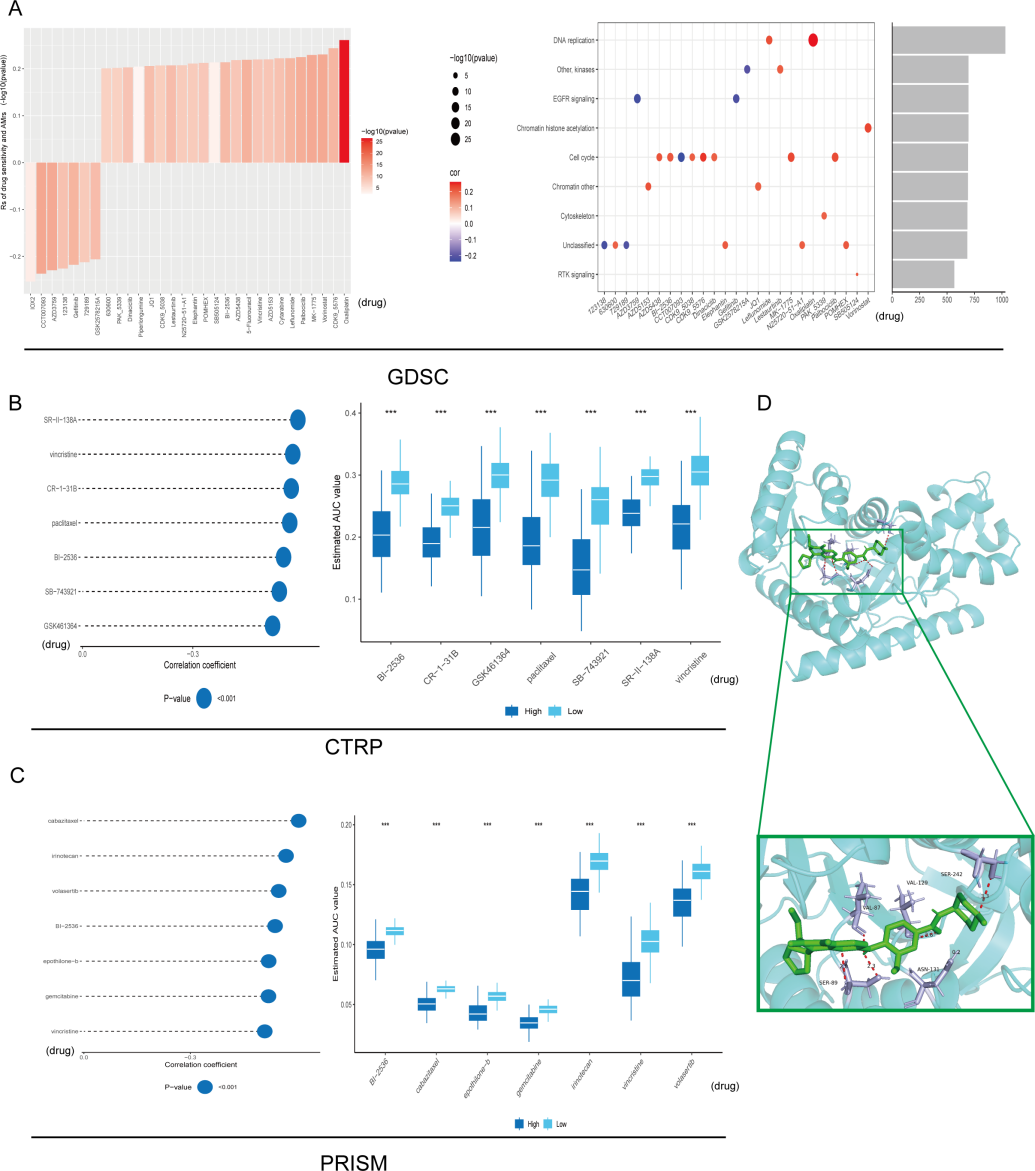
Drug sensitivity analysis. Signaling pathways and prospective therapeutic objectives associated with 25 putative compounds identified within the GDSC repository **(A)**. The correlation and difference between MDH1 and these predicted AUC values in CTRP **(B)** and PRISM **(C)** databases were explored, respectively. Autodock between BI-2536 and MDH1 (D). *** P< 0.001.

Across 1,837 compounds curated from PRISM, CTRP and GDSC, high MDH1 expression consistently predicted enhanced sensitivity to BI-2536 (Spearman r < –0.30, p < 0.05); oxaliplatin and leflunomide emerged as the strongest DNA-replication inhibitors in this panel (**Figures 8A–C**). To examine the underlying binding mode, we docked BI-2536 into the AlphaFold3-derived MDH1 structure, revealing ΔG = –9.02 kcal mol^−^¹ and key hydrogen bonds with Val87, Ser89, Val129 and Ser242 (**Figure 8D**). Subsequent 100-ns molecular-dynamics simulations at pH 7.2 confirmed complex stability (RMSD ≈ 0.1–0.25 nm), reduced RMSF in the binding pocket, persistent H-bonding and compact SASA (**Supplementary Figures 14A–E**). Thus, computational and pharmacogenomic data converge on BI-2536 as a high-affinity MDH1-directed agent.

To accelerate the clinical translation of MDH1 as a predictive biomarker, we next interrogated whether MDH1 expression levels modulate drug sensitivity by comparing the IC50 values of standard-of-care antineoplastic agents across lung-cancer cell lines stratified into MDH1-high and MDH1-low cohorts. Elevated MDH1 was associated with heightened vulnerability to cisplatin, docetaxel, paclitaxel, vinblastine and vinorelbine, whereas lower MDH1 predicted superior sensitivity to erlotinib, gefitinib and gemcitabine (**Supplementary Figure S15A**).

### Functional analysis of MDH1 in LUAD

To gain a deeper understanding of the pan-cancer implications of MDH1, a comprehensive analysis was conducted using the CancerSEA database. In this analysis, MDH1 expression was found to be positively associated with processes such as cell cycle progression, DNA damage response, DNA repair, epithelial-mesenchymal transition (EMT), invasiveness, metastatic potential, and proliferation. In contrast, MDH1 expression showed an inverse correlation with apoptotic pathways, hypoxic conditions, and inflammatory responses in lung adenocarcinoma, as shown in **Supplementary Figure S16A**.

**Figure 9:**
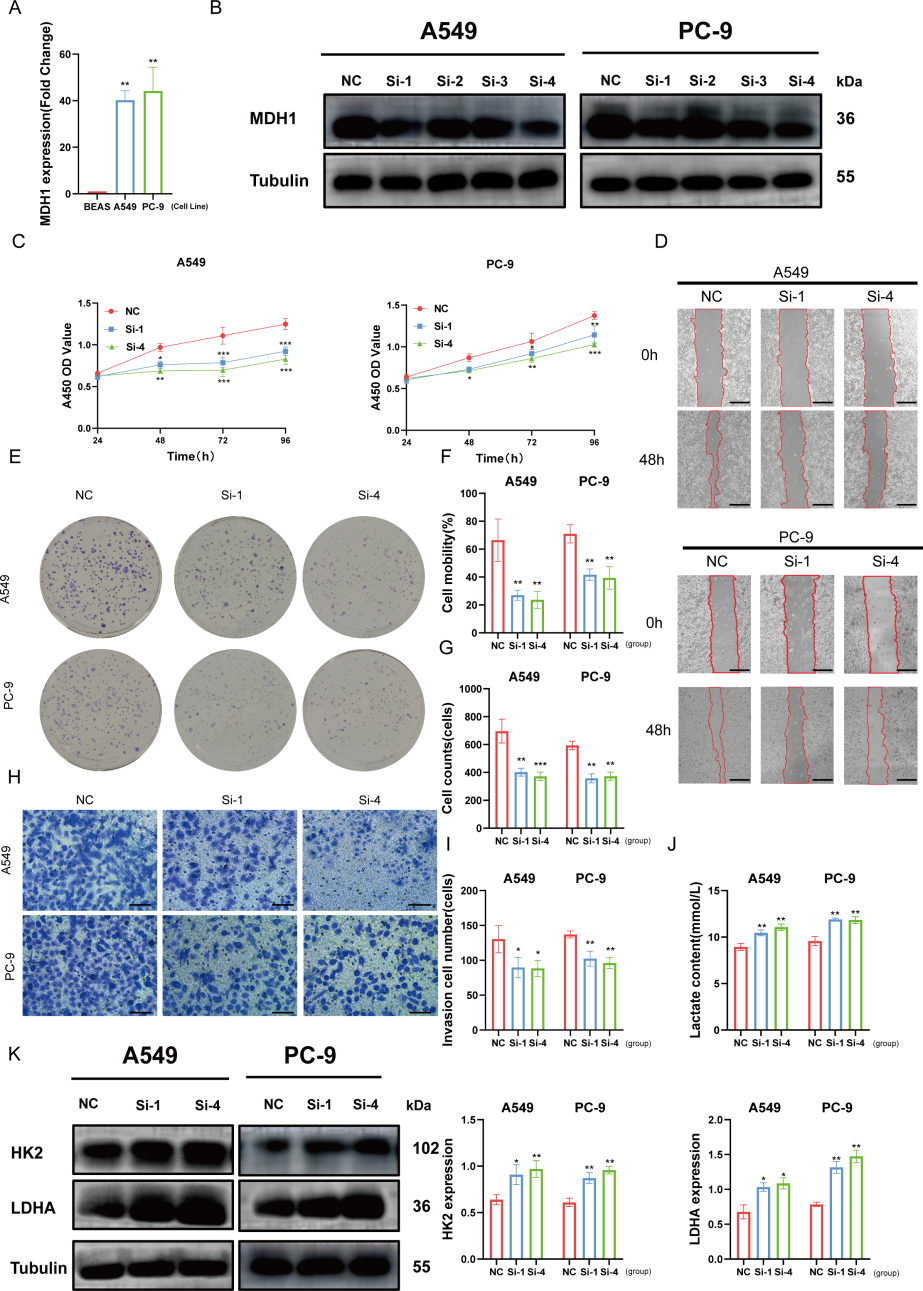
Effects of MDH1 on LUAD cell’s development. MDH1 RNA expression based on PCR analysis **(A)**. MDH1 protein expression based on western blot analysis **(B)**. The cell proliferative ability explored by CCK8 assay **(C)**. wound healing assays, Bars = 500 μm **(D, F)**. Colony formation assays **(E, G)**. Transwell assays, Bars = 100 μm **(H, I)**. The lactate content **(J)**. HK2, LDHA protein expression based on western blot analysis (K). *P < 0.05, **P < 0.01, ***P < 0.001.

To investigate the role of MDH1 in LUAD cell lines, we first demonstrated through PCR analysis that the expression levels of MDH1 were significantly higher in A549 and PC-9 cell lines compared to normal bronchial epithelial cells (**Figure 9A**). We then established MDH1 knockdown models by transfecting specific siRNAs. Following transfection with siRNAs targeting MDH1, WB analysis was conducted to assess knockdown efficiency. The results showed a significant reduction in MDH1 mRNA expression in A549 and PC-9 cells transfected with siRNA-MDH1–1 and siRNA-MDH1-4, compared to the control group (**Figure 9B**). These siRNA-transfected A549 and PC-9 cells were then used for further experiments.

The marked reduction in cell viability in both A549 and PC-9 cells transfected with si-MDH1, compared to the control group, between 48 and 96 hours post-transfection was demonstrated by CCK8 assays (**Figure 9C**). In order to assess the effect of MDH1 knockdown on cell proliferation, colony formation assays were conducted. These assays revealed a significant reduction in the number of colonies formed by A549 and PC-9 cells after MDH1 silencing (**Figures 9E, G**).

In addition, wound healing and transwell assays were utilized to evaluate the impact of MDH1 knockdown on cell migration. These experiments showed that silencing MDH1 significantly impaired the migratory capacity of LUAD cells (**Figures 9D, F, H, I**). Recent studies have indicated a potential role for MDH1 in hypoxia and glycolysis, but its function in LUAD remains unclear (54). Functional enrichment analysis has revealed that MDH1 is positively correlated with lactate metabolism in the vast majority of tumors (**Supplementary Figure S16B**). As expected, MDH1 knockdown increased lactate accumulation in culture supernatants (**Figure 9J**). Moreover, WB was utilized to examine the expression of glycolysis-related genes, and we found that MDH1 knockdown promoted glycolytic activity in LUAD cells (**Figure 9K**).

### MDH1 serves as a marker for infiltrating macrophage in tumor

**Figure 10:**
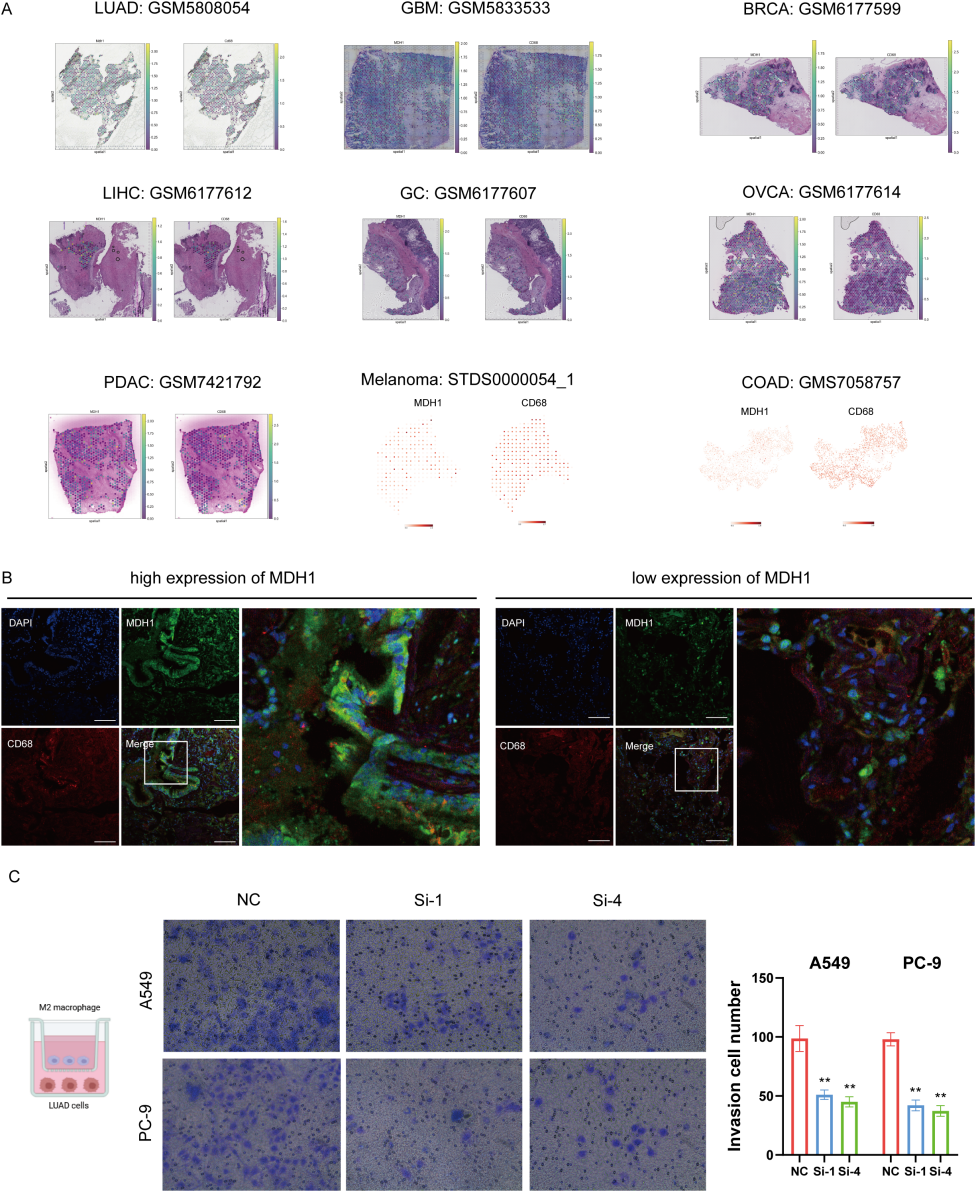
MDH1 is positive correlated with macrophage infiltration. Colocalization analysis of MDH1 and CD68 based on a pan-cancer spatial transcriptome cohort **(A)**. The immunofluorescence images demonstrate elevated protein levels of MDH1 and CD68. Bars = 100 μm **(B)**. Infiltration of M2 macrophage in A549 and PC-9 cells. Bar = 100 mm (C). ** P <0.01.

To further investigate the spatial relationship between MDH1 and the macrophage marker CD68, we used a pan-cancer spatial transcriptomics dataset. MDH1 exhibited a spatial distribution pattern that closely matched CD68, particularly in LUAD tissues (10A, **Supplementary Figures S8D** and **S17A**). Additionally, IF staining revealed elevated CD68 protein expression in regions with high MDH1 expression, while areas with low MDH1 expression showed reduced CD68 levels (**Figure 10B**). The coexpression of MDH1 and CD68 in LUAD tissues supported our previous findings. Based on this, we further evaluated the impact of MDH1 on M2 macrophages, observing a significant reduction in M2 macrophage infiltration in LUAD cells following MDH1 knockdown (**Figure 10C**).

To clarify whether MDH1-regulated macrophage function depends on lactate production, we co-cultured THP-1-derived macrophages with LUAD cells in which MDH1 had been knocked down or left intact. ELISA quantification of IL-10 and TNF-α in the conditioned medium revealed that MDH1 depletion significantly impaired M2 polarization (**Supplementary Figure S18A**). Importantly, pharmacologic inhibition of lactate (Fx11 (55)) failed to reverse this effect, indicating that MDH1 deficiency drives macrophages toward an M1 phenotype through a lactate-independent mechanism.

### Reducing MDH1 expression makes LUAD cells more sensitive to BI-2536

Our research revealed a positive correlation between MDH1 expression and the IC50 of BI-2536, indicating that elevated levels of MDH1 might be involved in fostering BI-2536 resistance in cancer cells. Additionally, the CCK8 assay reveal that diminishing the expression of MDH1 can enhance the susceptibility of LUAD cells to BI-2536 treatment (**Supplementary Figures S19A, B**).

To confirm the molecular interaction between MDH1 and BI-2536, we performed CETSA. The ligand-induced thermal stabilization of MDH1 in A549 cells verified their binding within the cellular context (**Supplementary Figure S19C**).

## Discussion

MDH1, a key enzyme in the MAS that operates in an NAD(H)-dependent manner, plays a crucial role in maintaining cellular redox balance. Disruptions in the MAS can cause imbalances in the cellular redox environment, promoting uncontrolled cell proliferation and survival. In the context of metabolic disorders, impaired activity of this shuttle can increase oxidative stress and inflammatory responses, further disrupting cellular metabolic homeostasis, as referenced in the literature (18).

To delve into the potential role of MDH1 in oncogenesis, an initial evaluation was undertaken by scrutinizing MDH1 expression across a spectrum of 33 distinct tumor types. Notably, mRNA levels of MDH1 were observed to be markedly elevated in various cancer types, including LUAD, when compared to their healthy counterparts. Following this, a suite of survival analyses was executed, revealing that patients with heightened MDH1 expression experienced poorer prognoses in LUAD, KICH, LAML, UVM, and CHOL. These findings are corroborated in part by existing evidence, which indicates that MDH1 sustains the levels of serine-threonine kinase ULK1, a critical initiator of autophagy, thereby enhancing cell survival and tumor progression in PAAD (9). Moreover, we have demonstrated that MDH1 expression is a risk prognostic factor for LUAD using 40 public LUAD datasets. Regardless of the prognostic model utilized, the outcomes of this investigation have established MDH1 as a neoplastic biomarker for the prognostication of LUAD. Subsequent analyses revealed a correlation between MDH1 expression and various clinicopathological parameters, including tumor stage, therapeutic responsiveness, and T and N classifications in LUAD. These factors were collectively associated with adverse survival outcomes for patients with LUAD. In conclusion, the conducted analyses suggest that MDH1 may serve as a prospective prognostic biomarker for a range of cancers, with particular relevance to LUAD.

A growing corpus of studies has unraveled associations between genetic alterations and diverse aspects of oncogenesis, containing tumor progression, metastatic spread, immune evasion, and therapeutic response (56–60). Our findings suggest that mutations in the MDH1 gene are notably frequent in LUAD and may have a detrimental impact on affected individuals. Specifically, the overexpression of MDH1 was significantly correlated with TMB and MSI across multiple cancer types. In LUAD, we observed a high frequency of MDH1 gene amplification. Additionally, a high mutation rate of PPP3CA was associated with low MDH1 expression, suggesting its relevance to bortezomib resistance (61). KRAS, a common oncogenic driver in LUAD (62), was positively linked to MDH1 expression. Marta Román et al. reported that KRAS-mutant patients show significant clinical benefit from PD-1 inhibitors (63). Furthermore, within the MDH1-low LUAD cohort, we observed a mutually exclusive occurrence of TP53 and KRAS mutations, accompanied by an increased somatic mutation rate. This molecular profile appears to be detrimental to the efficacy of ICIs (63, 64).

According to clinical consensus, DDR alterations, higher HRR, DNMT, and MMR are correlated with TMB and associated with greater clinical benefit following immunotherapy in LUAD (65–68). Moreover, DDR alterations, through genomic instability, may lead to a higher burden of non-synonymous mutations and immune escape (66). These characteristics were consistently observed in LUAD patients with elevated MDH1 expression. In a comprehensive analysis of 19 immunotherapy cohorts and a pan-cancer single-cell immunotherapy dataset, a significant finding was the elevated expression of MDH1 in patients who were sensitive to immunotherapy. This correlation suggests that MDH1 could serve as a potential predictive biomarker for immunotherapy response, highlighting the need for further investigation into its mechanistic role in treatment sensitivity.

Thus far, MDH1 research in oncology has predominantly concentrated on metabolic activity (24, 54, 69, 70). The TME, particularly its immune component, has been largely overlooked. Our study, for the first time, provides a comprehensive elucidation of MDH1’s impact on the TME. Our study establishes a basis for future in-depth investigations into the carcinogenic role of MDH1, highlighting the need for further research in this field. The scRNA-seq and functional enrichment analyses reveal that MDH1 is primarily expressed in macrophages and cancer cells, with a strong positive correlation to the proliferation and metastatic potential of LUAD. These results indicate that MDH1 functions as an oncogenic factor in the progression of various carcinomas, with LUAD being a prominent example. Furthermore, MDH1 plays a key role in lactate metabolism via the modulation of HK2 and LDHA expression. Although high glycolytic flux is generally associated with an immunosuppressive tumor milieu, our data uncover a previously unappreciated dissociation between lactate abundance and macrophage fate. Population-level analyses (TCGA) show that MDH1-low tumors coincide with a C3 immune-active signature, yet functional assays demonstrate that LDH inhibition neither rescues M2 polarization. Instead, genetic MDH1 loss alone is sufficient to redirect THP-1-derived M2 macrophages toward an M1 phenotype and to impair their trans-well migration. These findings indicate that MDH1 orchestrates macrophage behavior through a lactate-independent, cell-autonomous or contact-dependent axis, while the systemic enrichment of C3 in glycolytic tumors likely reflects collateral activation of other immune subsets (e.g., CD8^+^ T cells) rather than a direct lactate-driven immunosuppressive circuit. Consequently, therapeutic strategies targeting MDH1 may simultaneously blunt tumor glycolysis and re-educate TAMs without the need to neutralize lactate itself. Accumulated evidence has revealed the underlying impact of lactate in cancer immunotherapy. A high-level lactate microenvironment reduces tumor cells’ sensitivity to immunotherapy (71–73), which is consistent with our conclusion that the high-expression MDH1 group is more sensitive to immunotherapy.

TME, composed of immune cells, cancer cells, and stromal components, plays a crucial role in the progression, relapse, and chemoresistance of cancers (74, 75). A deeper understanding of the regulatory mechanisms within the TME could significantly improve therapeutic efficacy and support the development of novel treatment strategies (76). Our studies highlights that MDH1 is expressed in macrophages and epithelial cells revealed by single-cell analysis. However, there has been limited research on the mechanisms through which MDH1 influences the immune microenvironment. In our study, immunofluorescence analysis of human tissue samples demonstrated that MDH1 is enriched in macrophage regions. Moreover, our findings unraveled that MDH1 promotes the invasive behavior of these macrophages.

Further research has shown a significant positive association between MDH1 expression levels and the infiltration of MDSCs in the TME. Additionally, elevated MDH1 expression is linked to the exacerbation of CTL dysfunction. A substantial body of evidence indicates that MDSCs contribute to the formation of an immunosuppressive environment, which facilitates tumor angiogenesis, invasion, and metastasis (77–79). Future research should focus on elucidating the role of MDH1 in the immune microenvironment, with this study providing a foundational direction for such investigations.

Our observations indicate a positive correlation between elevated MDH1 levels and increased immune cell infiltration, activation of CD8+ T cells, and enhanced interferon-gamma (IFN-γ) response in lung adenocarcinoma (LUAD). Additionally, in the cohort with high MDH1 expression, we observed a notable upregulation of immune checkpoint molecules (LAG3, PD-1, LILRB2, and LILRB4), along with increased potential for cytolytic activity.

Analysis of 19 immunotherapy cohorts revealed that patients with high MDH1 expression may be more responsive to immunotherapeutic interventions. This conclusion was further supported by data from a pan-cancer single-cell immunotherapy cohort, which unveiled potential underlying mechanisms. This research highlights future efforts to overcome immunotherapy resistance in LUAD.

Given the heterogeneity of LUAD, we posit that a three-factor classifier combining MDH1 expression, PD-L1 status, and tumor mutational burden may outperform any single marker in predicting immunotherapy response. Prospective validation of this panel is underway. Furthermore, the ubiquitous expression of MDH1 in normal tissues raises concerns about on-target systemic toxicity if direct enzymatic inhibitors were pursued. Future work should therefore explore tumor-preferential delivery strategies—such as antibody–drug conjugates or PROTAC-mediated degradation—rather than classical small-molecule antagonists. In addition, synthetic-lethal combinations (e.g., pairing MDH1 blockade with oxidative-stress inducers) warrant pre-clinical evaluation to exploit metabolic vulnerabilities unique to cancer cells while sparing normal tissues.

It is important to acknowledge the limitations of our study. Our findings are primarily based on large-scale data analyses, which inherently limit the scope of our conclusions. Although initial insights into the involvement of MDH1 in cancer development and the tumor microenvironment have been gleaned from computational biology approaches, further experimental research in both cellular and whole-organism models is crucial for a more profound elucidation of MDH1’s physiological mechanisms. Moreover, our composite model awaits validation in prospective immunotherapy cohorts with standardized PD-L1 IHC and whole-exome sequencing. Additionally, the retrospective nature of TCGA limits causal inferences; hence, the clinical utility of the MDH1-containing panel should be confirmed in randomized trials.

## Conclusion

To summarize, the research findings indicate a consistent elevation of MDH1 expression across a range of cancers, with a strong correlation to poor oncological outcomes. The complex interaction between MDH1 and macrophages in the tumor microenvironment, along with its impact on the efficacy of immunotherapies in various cancers, is particularly significant. These findings position MDH1 as a underlying biomarker for immunotherapy, aiding in the identification of cancer patients who are more likely to respond to such treatments. We have further confirmed the role of MDH1 as an oncogenic factor in LUAD. Therefore, we propose considering MDH1 as a potential prognostic biomarker and a promising predictor of immunotherapy sensitivity in various malignancies. However, our current understanding of MDH1 remains limited, and future research is needed to explore its additional functional roles, underlying mechanisms, and potential as a therapeutic target.

## Data Availability

The datasets presented in this study can be found in online repositories. The names of the repository/repositories and accession number(s) can be found in the article/**Supplementary Material**.

## References

[1] Siegel RL Miller KD Fuchs HE Jemal A. Cancer statistics, 2021. CA Cancer J Clin. (2021) 71:7–33. doi: 10.3322/caac.21654, PMID: 33433946

[2] Sung H Ferlay J Siegel RL Laversanne M Soerjomataram I Jemal A. Global cancer statistics 2020: GLOBOCAN estimates of incidence and mortality worldwide for 36 cancers in 185 countries. CA Cancer J Clin. (2021) 71:209–49. doi: 10.3322/caac.21660, PMID: 33538338

[3] Blum A Wang P Zenklusen JC. SnapShot: TCGA-analyzed tumors. Cell. (2018) 173:530. doi: 10.1016/j.cell.2018.03.059, PMID: 29625059

[4] Manochkumar J Cherukuri AK Kumar RS Almansour AI Ramamoorthy S Efferth T. A critical review of machine-learning for “multi-omics” marine metabolite datasets. Comput Biol Med. (2023) 165:107425. doi: 10.1016/j.compbiomed.2023.107425, PMID: 37696182

[5] Ouyang D Liang Y Li L Ai N Lu S Yu M. Integration of multi-omics data using adaptive graph learning and attention mechanism for patient classification and biomarker identification. Comput Biol Med. (2023) 164:107303. doi: 10.1016/j.compbiomed.2023.107303, PMID: 37586201

[6] Su H Zhao D Elmannai H Heidari AA Bourouis S Wu Z. Multilevel threshold image segmentation for COVID-19 chest radiography: A framework using horizontal and vertical multiverse optimization. Comput Biol Med. (2022) 146:105618. doi: 10.1016/j.compbiomed.2022.105618, PMID: 35690477 PMC9113963

[7] Jiang X Ding Y Liu M Wang Y Li Y Wu Z. BiFTransNet: A unified and simultaneous segmentation network for gastrointestinal images of CT & MRI. Comput Biol Med. (2023) 165:107326. doi: 10.1016/j.compbiomed.2023.107326, PMID: 37619324

[8] Xie J Deng X Xie Y Zhu H Liu P Deng W. Multi-omics analysis of disulfidptosis regulators and therapeutic potential reveals glycogen synthase 1 as a disulfidptosis triggering target for triple-negative breast cancer. MedComm (2020). (2024) 5:e502. doi: 10.1002/mco2.502, PMID: 38420162 PMC10901283

[9] New M Van Acker T Sakamaki JI Jiang M Saunders RE Long J. MDH1 and MPP7 regulate autophagy in pancreatic ductal adenocarcinoma. Cancer Res. (2019) 79:1884–98. doi: 10.1158/0008-5472.CAN-18-2553, PMID: 30765601 PMC6522344

[10] Hanse EA Ruan C Kachman M Wang D Lowman XH Kelekar A. Cytosolic malate dehydrogenase activity helps support glycolysis in actively proliferating cells and cancer. Oncogene. (2017) 36:3915–24. doi: 10.1038/onc.2017.36, PMID: 28263970 PMC5501748

[11] Olguin-Albuerne M Moran J. Redox signaling mechanisms in nervous system development. Antioxid Redox Signal. (2018) 28:1603–25. doi: 10.1089/ars.2017.7284, PMID: 28817955

[12] Webb LE Hill EJ Banaszak LJ. Conformation of nicotinamide adenine dinucleotide bound to cytoplasmic malate dehydrogenase. Biochemistry. (1973) 12:5101–9. doi: 10.1021/bi00749a013, PMID: 4366080

[13] Joh T Takeshima H Tsuzuki T Setoyama C Shimada K Tanase S. Cloning and sequence analysis of cDNAs encoding mammalian cytosolic malate dehydrogenase. Comparison of the amino acid sequences of mammalian and bacterial malate dehydrogenase. J Biol Chem. (1987) 262:15127–31. doi: 10.1016/S0021-9258(18)48147-1, PMID: 3312200

[14] Tanaka T Inazawa J Nakamura Y. Molecular cloning and mapping of a human cDNA for cytosolic malate dehydrogenase (MDH1). Genomics. (1996) 32:128–30. doi: 10.1006/geno.1996.0087, PMID: 8786100

[15] Lo AS Liew CT Ngai SM Tsui SK Fung KP Lee CY. Developmental regulation and cellular distribution of human cytosolic malate dehydrogenase (MDH1). J Cell Biochem. (2005) 94:763–73. doi: 10.1002/jcb.20343, PMID: 15565635

[16] Gaude E Schmidt C Gammage PA Dugourd A Blacker T Chew SP. NADH shuttling couples cytosolic reductive carboxylation of glutamine with glycolysis in cells with mitochondrial dysfunction. Mol Cell. (2018) 69:581–93 e7. doi: 10.1016/j.molcel.2018.01.034, PMID: 29452638 PMC5823973

[17] Wang L Wang D Zeng X Zhang Q Wu H Liu J. Exploration of spatial heterogeneity of tumor microenvironment in nasopharyngeal carcinoma via transcriptional digital spatial profiling. Int J Biol Sci. (2023) 19:2256–69. doi: 10.7150/ijbs.74653, PMID: 37151882 PMC10158028

[18] Parente AD Bolland DE Huisinga KL Provost JJ. Physiology of malate dehydrogenase and how dysregulation leads to disease. Essays Biochem. (2024) 68:121–34. doi: 10.1042/EBC20230085, PMID: 38962852

[19] Haberman A Peterson CN. Genetics of MDH in humans. Essays Biochem. (2024) 68:107–19. doi: 10.1042/EBC20230078, PMID: 39037390

[20] Zhang B Tornmalm J Widengren J Vakifahmetoglu-Norberg H Norberg E. Characterization of the role of the malate dehydrogenases to lung tumor cell survival. J Cancer. (2017) 8:2088–96. doi: 10.7150/jca.19373, PMID: 28819410 PMC5559971

[21] Godesi S Han JR Kim JK Kwak DI Lee J Nada H. Design, synthesis and biological evaluation of novel MDH inhibitors targeting tumor microenvironment. Pharm (Basel). (2023) 16(5):683-717. doi: 10.3390/ph16050683, PMID: 37242466 PMC10263210

[22] Lee SM Dho SH Ju SK Maeng JS Kim JY Kwon KS. Cytosolic malate dehydrogenase regulates senescence in human fibroblasts. Biogerontology. (2012) 13:525–36. doi: 10.1007/s10522-012-9397-0, PMID: 22971926

[23] Lee SM Kim JH Cho EJ Youn HD. A nucleocytoplasmic malate dehydrogenase regulates p53 transcriptional activity in response to metabolic stress. Cell Death Differ. (2009) 16:738–48. doi: 10.1038/cdd.2009.5, PMID: 19229245

[24] Zhu Q Zhou H Wu L Lai Z Geng D Yang W. O-GlcNAcylation promotes pancreatic tumor growth by regulating malate dehydrogenase 1. Nat Chem Biol. (2022) 18:1087–95. doi: 10.1038/s41589-022-01085-5, PMID: 35879546

[25] Carlino MS Larkin J Long GV. Immune checkpoint inhibitors in melanoma. Lancet. (2021) 398:1002–14. doi: 10.1016/S0140-6736(21)01206-X, PMID: 34509219

[26] Kelly RJ Ajani JA Kuzdzal J Zander T Van Cutsem E Piessen G. Adjuvant nivolumab in resected esophageal or gastroesophageal junction cancer. N Engl J Med. (2021) 384:1191–203. doi: 10.1056/NEJMoa2032125, PMID: 33789008

[27] Postow MA Sidlow R Hellmann MD. Immune-related adverse events associated with immune checkpoint blockade. N Engl J Med. (2018) 378:158–68. doi: 10.1056/NEJMra1703481 29320654

[28] Ott PA Bang YJ Piha-Paul SA Razak ARA Bennouna J Soria JC. T-cell-inflamed gene-expression profile, programmed death ligand 1 expression, and tumor mutational burden predict efficacy in patients treated with pembrolizumab across 20 cancers: KEYNOTE-028. J Clin Oncol. (2019) 37:318–27. doi: 10.1200/JCO.2018.78.2276, PMID: 30557521

[29] Overman MJ McDermott R Leach JL Lonardi S Lenz HJ Morse MA. Nivolumab in patients with metastatic DNA mismatch repair-deficient or microsatellite instability-high colorectal cancer (CheckMate 142): an open-label, multicentre, phase 2 study. Lancet Oncol. (2017) 18:1182–91. doi: 10.1016/S1470-2045(17)30422-9, PMID: 28734759 PMC6207072

[30] Tumeh PC Harview CL Yearley JH Shintaku IP Taylor EJ Robert L. PD-1 blockade induces responses by inhibiting adaptive immune resistance. Nature. (2014) 515:568–71. doi: 10.1038/nature13954, PMID: 25428505 PMC4246418

[31] Gao J Aksoy BA Dogrusoz U Dresdner G Gross B Sumer SO. Integrative analysis of complex cancer genomics and clinical profiles using the cBioPortal. Sci Signal. (2013) 6:pl1. doi: 10.1126/scisignal.2004088, PMID: 23550210 PMC4160307

[32] Chen Z Luo Z Zhang D Li H Liu X Zhu K. TIGER: A web portal of tumor immunotherapy gene expression resource. Genomics Proteomics Bioinf. (2023) 21:337–48. doi: 10.1016/j.gpb.2022.08.004, PMID: 36049666 PMC10626175

[33] Wolf FA Angerer P Theis FJ. SCANPY: large-scale single-cell gene expression data analysis. Genome Biol. (2018) 19:15. doi: 10.1186/s13059-017-1382-0, PMID: 29409532 PMC5802054

[34] Charoentong P Finotello F Angelova M Mayer C Efremova M Rieder D. Pan-cancer immunogenomic analyses reveal genotype-immunophenotype relationships and predictors of response to checkpoint blockade. Cell Rep. (2017) 18:248–62. doi: 10.1016/j.celrep.2016.12.019, PMID: 28052254

[35] Zhang Y Yao X Zhou H Wu X Tian J Zeng J. OncoSplicing: an updated database for clinically relevant alternative splicing in 33 human cancers. Nucleic Acids Res. (2022) 50:D1340–D7. doi: 10.1093/nar/gkab851, PMID: 34554251 PMC8728274

[36] Yu C Mannan AM Yvone GM Ross KN Zhang YL Marton MA. High-throughput identification of genotype-specific cancer vulnerabilities in mixtures of barcoded tumor cell lines. Nat Biotechnol. (2016) 34:419–23. doi: 10.1038/nbt.3460, PMID: 26928769 PMC5508574

[37] Basu A Bodycombe NE Cheah JH Price EV Liu K Schaefer GI. An interactive resource to identify cancer genetic and lineage dependencies targeted by small molecules. Cell. (2013) 154:1151–61. doi: 10.1016/j.cell.2013.08.003, PMID: 23993102 PMC3954635

[38] Yang W Soares J Greninger P Edelman EJ Lightfoot H Forbes S. Genomics of Drug Sensitivity in Cancer (GDSC): a resource for therapeutic biomarker discovery in cancer cells. Nucleic Acids Res. (2013) 41:D955–61. doi: 10.1093/nar/gks1111, PMID: 23180760 PMC3531057

[39] Graham F. Daily briefing: AlphaFold3 is now open source. Nature. (2024). doi: 10.1038/d41586-024-03728-0, PMID: 39543293

[40] Yang X Wang S Tang Y Ying Y Zhu Y Chen C. Food additive salicylates inhibit human and rat placental 3β-hydroxysteroid dehydrogenase: 3D-QSAR and in silico analysis. Chem Biol Interact. (2024) 402:111203. doi: 10.1016/j.cbi.2024.111203, PMID: 39159849

[41] Van Der Spoel D Lindahl E Hess B Groenhof G Mark AE Berendsen HJ. GROMACS: fast, flexible, and free. J Comput Chem. (2005) 26:1701–18. doi: 10.1002/jcc.20291, PMID: 16211538

[42] Dai YW Wu ZX Cheng Y Wu HD Chen JW Lv LX. Formosanin C inhibits triple-negative breast cancer progression by suppressing the phosphorylation of STAT3 and the polarization of M2 macrophages. Breast Cancer Res Treat. (2025) 211(1):71–89. doi: 10.1007/s10549-025-07623-8, PMID: 39953272

[43] Xiao H Chen C Yuan X Yang L Zheng Y Yuan J. Gingerenone A induces ferroptosis in colorectal cancer via targeting suppression of SLC7A11 signaling pathway. BioMed Pharmacother. (2024) 180:117529. doi: 10.1016/j.biopha.2024.117529, PMID: 39393329

[44] Garraway LA Lander ES. Lessons from the cancer genome. Cell. (2013) 153:17–37. doi: 10.1016/j.cell.2013.03.002, PMID: 23540688

[45] Torres CM Biran A Burney MJ Patel H Henser-Brownhill T Cohen AS. The linker histone H1.0 generates epigenetic and functional intratumor heterogeneity. Science. (2016) 353(6307):aaf1644. doi: 10.1126/science.aaf1644, PMID: 27708074 PMC5131846

[46] Chandrashekar DS Bashel B Balasubramanya SAH Creighton CJ Ponce-Rodriguez I Chakravarthi B. UALCAN: A portal for facilitating tumor subgroup gene expression and survival analyses. Neoplasia. (2017) 19:649–58. doi: 10.1016/j.neo.2017.05.002, PMID: 28732212 PMC5516091

[47] Bonnal SC Lopez-Oreja I Valcarcel J. Roles and mechanisms of alternative splicing in cancer - implications for care. Nat Rev Clin Oncol. (2020) 17:457–74. doi: 10.1038/s41571-020-0350-x, PMID: 32303702

[48] Teo MY Seier K Ostrovnaya I Regazzi AM Kania BE Moran MM. Alterations in DNA damage response and repair genes as potential marker of clinical benefit from PD-1/PD-L1 blockade in advanced urothelial cancers. J Clin Oncol. (2018) 36:1685–94. doi: 10.1200/JCO.2017.75.7740, PMID: 29489427 PMC6366295

[49] Jia L Wang T Zhao Y Zhang S Ba T Kuai X. Single-cell profiling of infiltrating B cells and tertiary lymphoid structures in the TME of gastric adenocarcinomas. Oncoimmunology. (2021) 10:1969767. doi: 10.1080/2162402X.2021.1969767, PMID: 34513317 PMC8425751

[50] Dai YW Wang WM Zhou X. Development of a CD8(+) T cell-based molecular classification for predicting prognosis and heterogeneity in triple-negative breast cancer by integrated analysis of single-cell and bulk RNA-sequencing. Heliyon. (2023) 9:e19798. doi: 10.1016/j.heliyon.2023.e19798, PMID: 37810147 PMC10559128

[51] Coulton A Murai J Qian D Thakkar K Lewis CE Litchfield K. Using a pan-cancer atlas to investigate tumour associated macrophages as regulators of immunotherapy response. Nat Commun. (2024) 15:5665. doi: 10.1038/s41467-024-49885-8, PMID: 38969631 PMC11226649

[52] Wu G Cheng H Yin J Zheng Y Shi H Pan B. NDRG1-driven lactate accumulation promotes lung adenocarcinoma progression through the induction of an immunosuppressive microenvironment. Adv Sci (Weinh). (2025) 20:e01238. doi: 10.1002/advs.202501238, PMID: 40539245 PMC12412559

[53] Wu Z Su R Dai Y Wu X Wu H Wang X. Deep pan-cancer analysis and multi-omics evidence reveal that ALG3 inhibits CD8(+) T cell infiltration by suppressing chemokine secretion and is associated with 5-fluorouracil sensitivity. Comput Biol Med. (2024) 177:108666. doi: 10.1016/j.compbiomed.2024.108666, PMID: 38820773

[54] Grimm F Asuaje A Jain A Silva Dos Santos M Kleinjung J Nunes PM. Metabolic priming by multiple enzyme systems supports glycolysis, HIF1alpha stabilisation, and human cancer cell survival in early hypoxia. EMBO J. (2024) 43:1545–69. doi: 10.1038/s44318-024-00065-w, PMID: 38485816 PMC11021510

[55] Rao K Zhang X Luo Y Xia Q Jin Y He J. Lactylation orchestrates ubiquitin-independent degradation of cGAS and promotes tumor growth. Cell Rep. (2025) 44:115441. doi: 10.1016/j.celrep.2025.115441, PMID: 40106438

[56] Berger MF Mardis ER. The emerging clinical relevance of genomics in cancer medicine. Nat Rev Clin Oncol. (2018) 15:353–65. doi: 10.1038/s41571-018-0002-6, PMID: 29599476 PMC6658089

[57] Cha S Lee E Won HH. Comprehensive characterization of distinct genetic alterations in metastatic breast cancer across various metastatic sites. NPJ Breast Cancer. (2021) 7:93. doi: 10.1038/s41523-021-00303-y, PMID: 34272397 PMC8285498

[58] Yang D Khan S Sun Y Hess K Shmulevich I Sood AK. Association of BRCA1 and BRCA2 mutations with survival, chemotherapy sensitivity, and gene mutator phenotype in patients with ovarian cancer. JAMA. (2011) 306:1557–65. doi: 10.1001/jama.2011.1456, PMID: 21990299 PMC4159096

[59] Zhu M Kim J Deng Q Ricciuti B Alessi JV Eglenen-Polat B. Loss of p53 and mutational heterogeneity drives immune resistance in an autochthonous mouse lung cancer model with high tumor mutational burden. Cancer Cell. (2023) 41:1731–48 e8. doi: 10.1016/j.ccell.2023.09.006, PMID: 37774698 PMC10693909

[60] Ricciuti B Elkrief A Alessi J Wang X Li Y Gupta H. Clinicopathologic, genomic, and immunophenotypic landscape of ATM mutations in non-small cell lung cancer. Clin Cancer Res. (2023) 29:2540–50. doi: 10.1158/1078-0432.CCR-22-3413, PMID: 37097610 PMC11031845

[61] Imai Y Ohta E Takeda S Sunamura S Ishibashi M Tamura H. Histone deacetylase inhibitor panobinostat induces calcineurin degradation in multiple myeloma. JCI Insight. (2016) 1:e85061. doi: 10.1172/jci.insight.85061, PMID: 27699258 PMC5033869

[62] Salgia R Pharaon R Mambetsariev I Nam A Sattler M. The improbable targeted therapy: KRAS as an emerging target in non-small cell lung cancer (NSCLC). Cell Rep Med. (2021) 2:100186. doi: 10.1016/j.xcrm.2020.100186, PMID: 33521700 PMC7817862

[63] Roman M Baraibar I Lopez I Nadal E Rolfo C Vicent S. KRAS oncogene in non-small cell lung cancer: clinical perspectives on the treatment of an old target. Mol Cancer. (2018) 17:33. doi: 10.1186/s12943-018-0789-x, PMID: 29455666 PMC5817724

[64] Dantoing E Piton N Salaun M Thiberville L Guisier F. Anti-PD1/PD-L1 immunotherapy for non-small cell lung cancer with actionable oncogenic driver mutations. Int J Mol Sci. (2021) 22 (12):6288. doi: 10.3390/ijms22126288, PMID: 34208111 PMC8230861

[65] Zhou Z Ding Z Yuan J Shen S Jian H Tan Q. Homologous recombination deficiency (HRD) can predict the therapeutic outcomes of immuno-neoadjuvant therapy in NSCLC patients. J Hematol Oncol. (2022) 15:62. doi: 10.1186/s13045-022-01283-7, PMID: 35585646 PMC9118717

[66] Bzura A Spicer JB Dulloo S Yap TA Fennell DA. Targeting DNA damage response deficiency in thoracic cancers. Drugs. (2024) 84:1025–33. doi: 10.1007/s40265-024-02066-9, PMID: 39001941

[67] Marzio A Kurz E Sahni JM Di Feo G Puccini J Jiang S. EMSY inhibits homologous recombination repair and the interferon response, promoting lung cancer immune evasion. Cell. (2022) 185:169–83 e19. doi: 10.1016/j.cell.2021.12.005, PMID: 34963055 PMC8751279

[68] Zheng X Lin J Xiong J Guan Y Lan B Li Y. SETD2 variation correlates with tumor mutational burden and MSI along with improved response to immunotherapy. BMC Cancer. (2023) 23:686. doi: 10.1186/s12885-023-10920-4, PMID: 37479966 PMC10360270

[69] Wang J Hong M Cheng Y Wang X Li D Chen G. Targeting c-Myc transactivation by LMNA inhibits tRNA processing essential for malate-aspartate shuttle and tumour progression. Clin Transl Med. (2024) 14:e1680. doi: 10.1002/ctm2.1680, PMID: 38769668 PMC11106511

[70] Tang D Zheng Y Wang G Sheng C Liu Z Wang B. HPV18 E6 inhibits α-ketoglutarate-induced pyroptosis of esophageal squamous cell carcinoma cells via the P53/MDH1/ROS/GSDMC pathway. FEBS Open Bio. (2023) 13:1522–35. doi: 10.1002/2211-5463.13666, PMID: 37402485 PMC10392054

[71] Chen J Huang Z Chen Y Tian H Chai P Shen Y. Lactate and lactylation in cancer. Signal Transduct Target Ther. (2025) 10:38. doi: 10.1038/s41392-024-02082-x, PMID: 39934144 PMC11814237

[72] Zha J Zhang J Lu J Zhang G Hua M Guo W. A review of lactate-lactylation in Malignancy: its potential in immunotherapy. Front Immunol. (2024) 15:1384948. doi: 10.3389/fimmu.2024.1384948, PMID: 38779665 PMC11109376

[73] Yu T Liu Z Tao Q Xu X Li X Li Y. Targeting tumor-intrinsic SLC16A3 to enhance anti-PD-1 efficacy via tumor immune microenvironment reprogramming. Cancer Lett. (2024) 589:216824. doi: 10.1016/j.canlet.2024.216824, PMID: 38522774

[74] Hinshaw DC Shevde LA. The tumor microenvironment innately modulates cancer progression. Cancer Res. (2019) 79:4557–66. doi: 10.1158/0008-5472.CAN-18-3962, PMID: 31350295 PMC6744958

[75] Wu T Dai Y. Tumor microenvironment and therapeutic response. Cancer Lett. (2017) 387:61–8. doi: 10.1016/j.canlet.2016.01.043, PMID: 26845449

[76] Pitt JM Marabelle A Eggermont A Soria JC Kroemer G Zitvogel L. Targeting the tumor microenvironment: removing obstruction to anticancer immune responses and immunotherapy. Ann Oncol. (2016) 27:1482–92. doi: 10.1093/annonc/mdw168, PMID: 27069014

[77] Bronte V Brandau S Chen SH Colombo MP Frey AB Greten TF. Recommendations for myeloid-derived suppressor cell nomenclature and characterization standards. Nat Commun. (2016) 7:12150. doi: 10.1038/ncomms12150, PMID: 27381735 PMC4935811

[78] Gabrilovich DI Nagaraj S. Myeloid-derived suppressor cells as regulators of the immune system. Nat Rev Immunol. (2009) 9:162–74. doi: 10.1038/nri2506, PMID: 19197294 PMC2828349

[79] Gabrilovich DI Ostrand-Rosenberg S Bronte V. Coordinated regulation of myeloid cells by tumours. Nat Rev Immunol. (2012) 12:253–68. doi: 10.1038/nri3175, PMID: 22437938 PMC3587148

