# Supplementary material for: Panoramic view of MDH1: driving cancer progression and shaping the tumor immune microenvironment
Source: Front Immunol. 2025 Aug 28;16:1631449. doi: 10.3389/fimmu.2025.1631449 (PMC12423056; doi:10.3389/fimmu.2025.1631449)
Supplement: Supplementary file 1 [file DataSheet1.docx]

**Supplement figure**

**
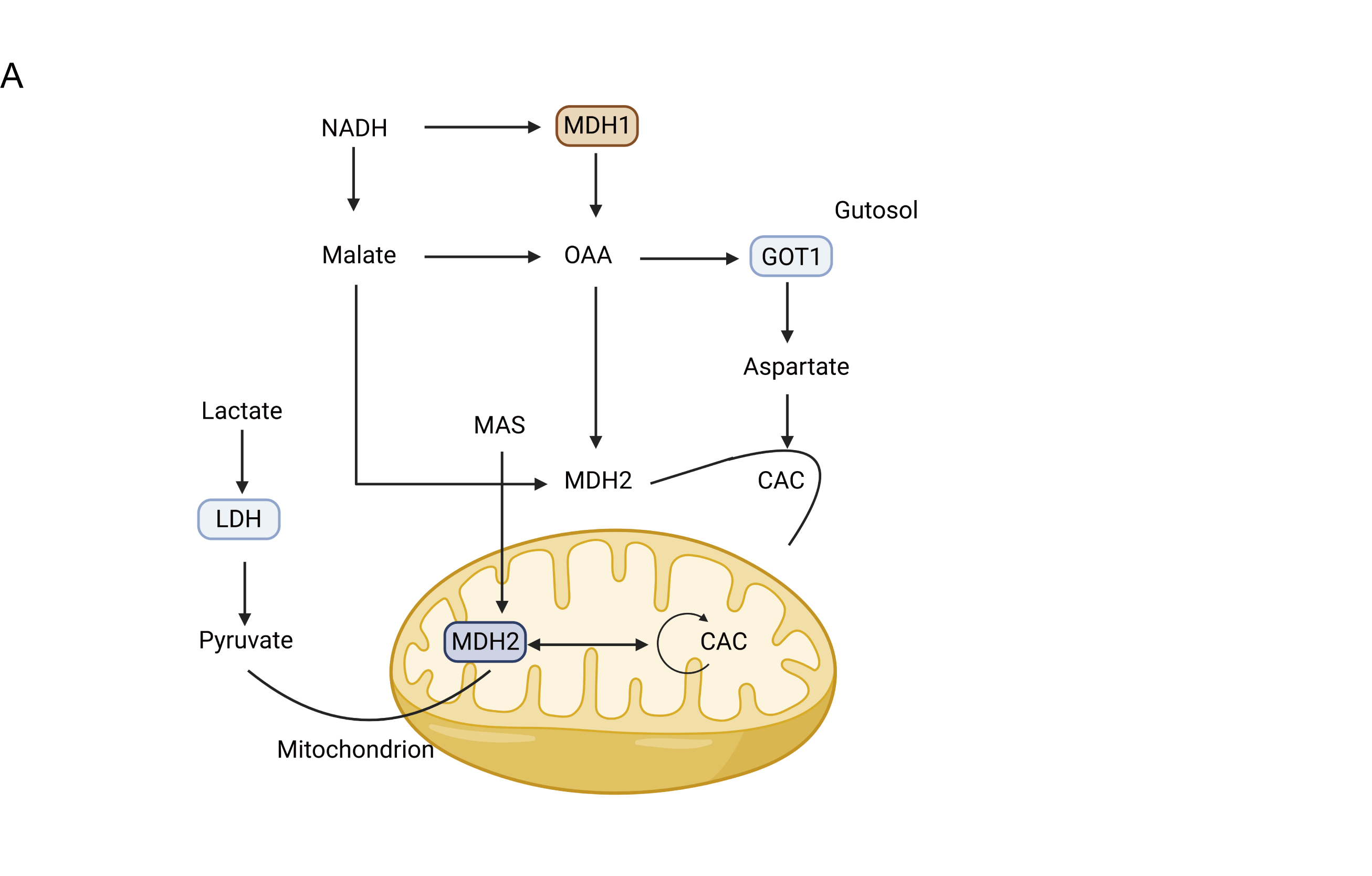
**

Supplementary Fig. 1

Schematic overview of the metabolic pathway(A). MAS, Malate–Aspartate Shuttle;
CAC, Citric Acid Cycle; LDH, Lactate Dehydrogenase Pathway; MDH1, Malate Dehydrogenase 1 (Cytosolic); MDH2, Malate Dehydrogenase 2 (Mitochondrial); GOT1, Glutamate Oxaloacetate Transaminase 1 (Cytosolic Aspartate Transaminase); OAA, Oxaloacetate; Malate, Malic Acid;


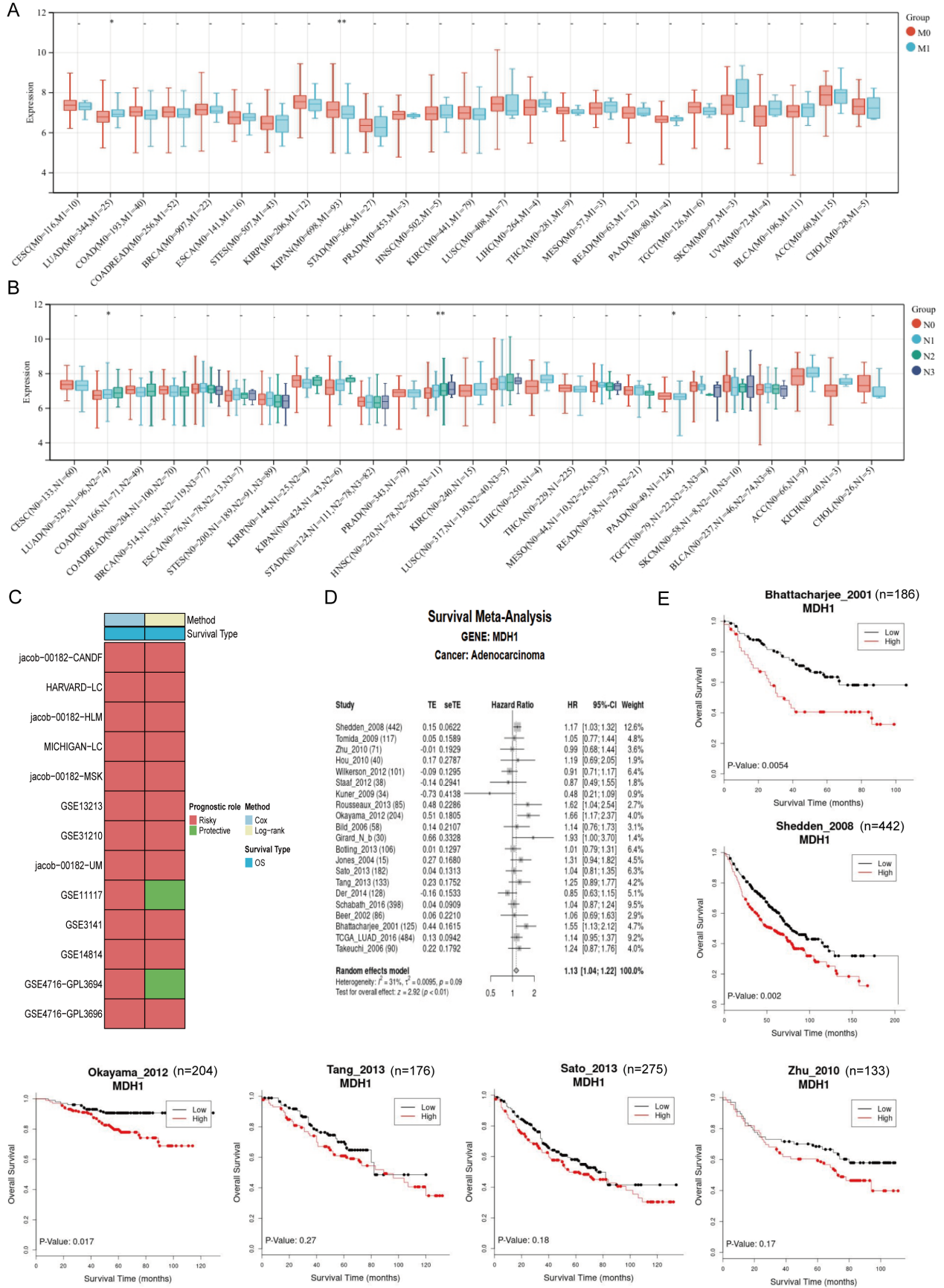


Supplementary Fig. 2

Analysis of clinical features of MDH1 in multiple-cancer cohorts. The correlation between MDH1 expression and N(A), M(B) in pan-cancer. The heapmap(C) and table(D) of survival analysis. K–M curves based on MDH1 expression(E).


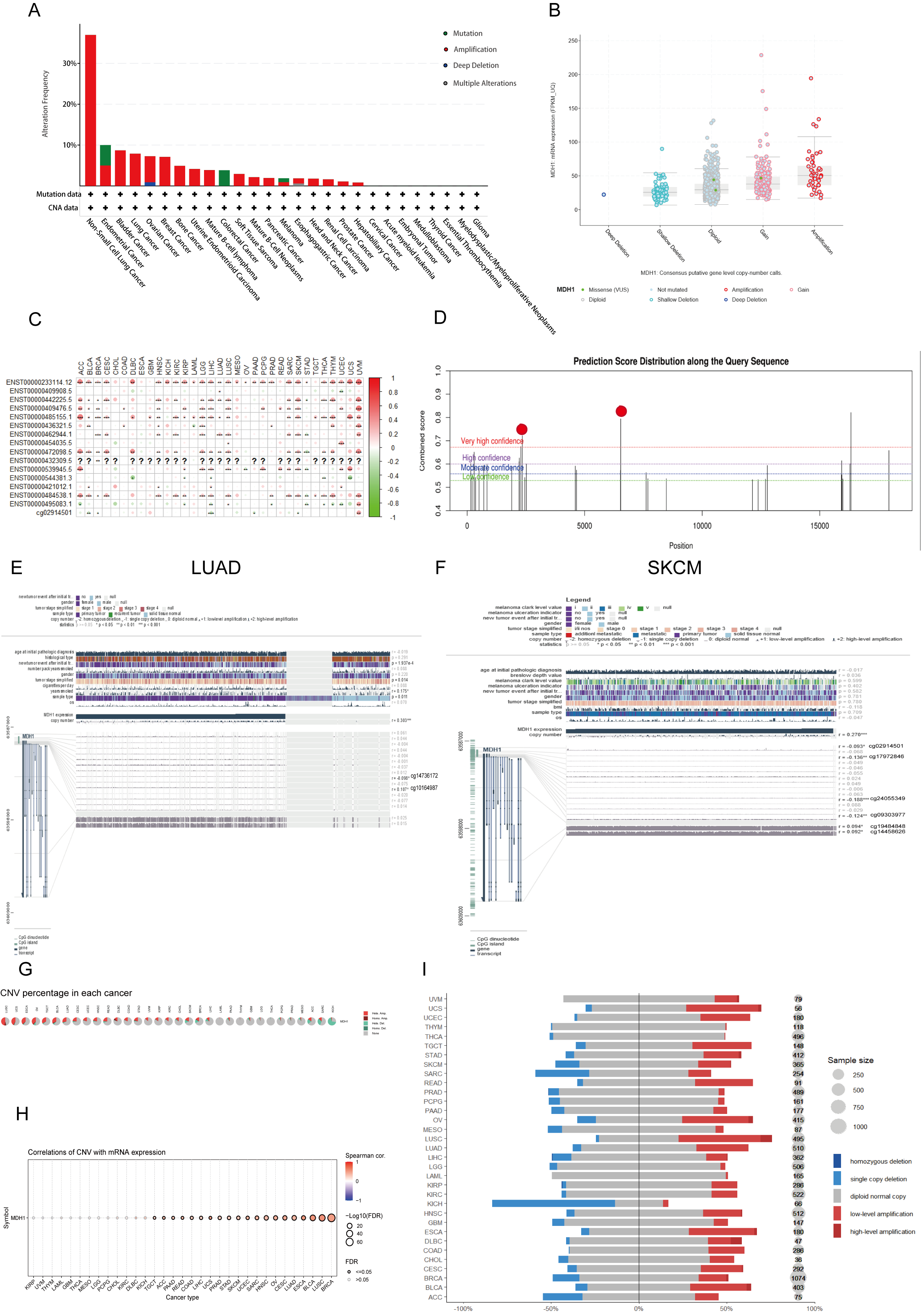


Supplementary Fig. 3

Genetic variations landscape of MDH1. MDH1 alteration frequencies (A). MDH1 expression in different mutation types (B). DNA methylation analysis of MDH1, based on TCGA and GTEx databases. Red and green squares represent upregulation and downregulation, respectively. The x-axis represents various cancer types, and the y-axis represents different DNA methylation probes. t-test, *P < 0.05, **P < 0.01, ****P < 0.0001 (C), alongside computational identification of m6A modification sites within the MDH1 sequence (D). The methylation sites within the MDH1 DNA sequence in LUAD (E). CNV profiles of MDH1 in pan-cancer (G). Correlations analysis between MDH1 and CNV(H). DNA copy number variation analysis in pan-cancers (I).


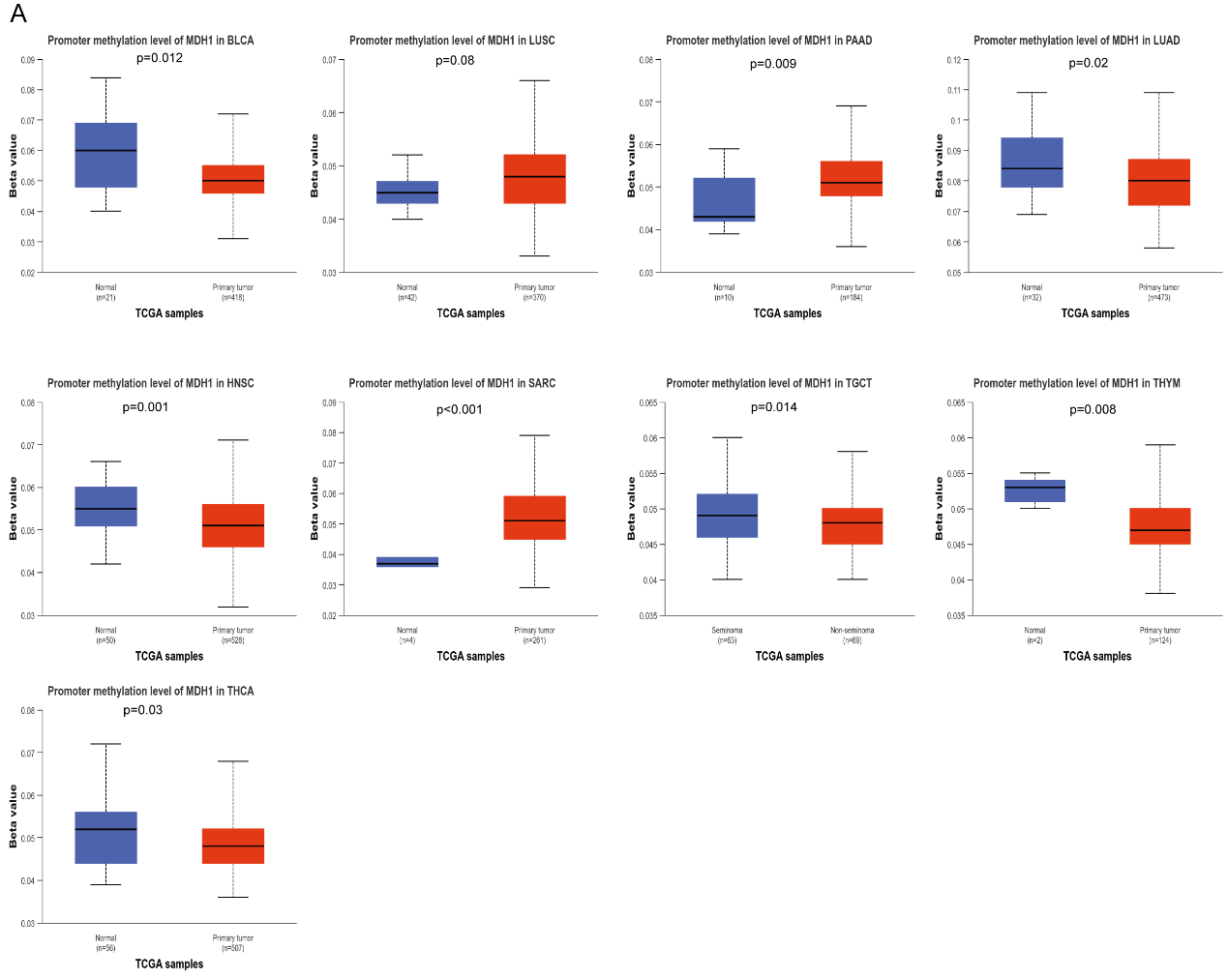


Supplementary Fig. 4

The differential analysis of promoter methylation levels of MDH1 in pan-cancer cohort(A).


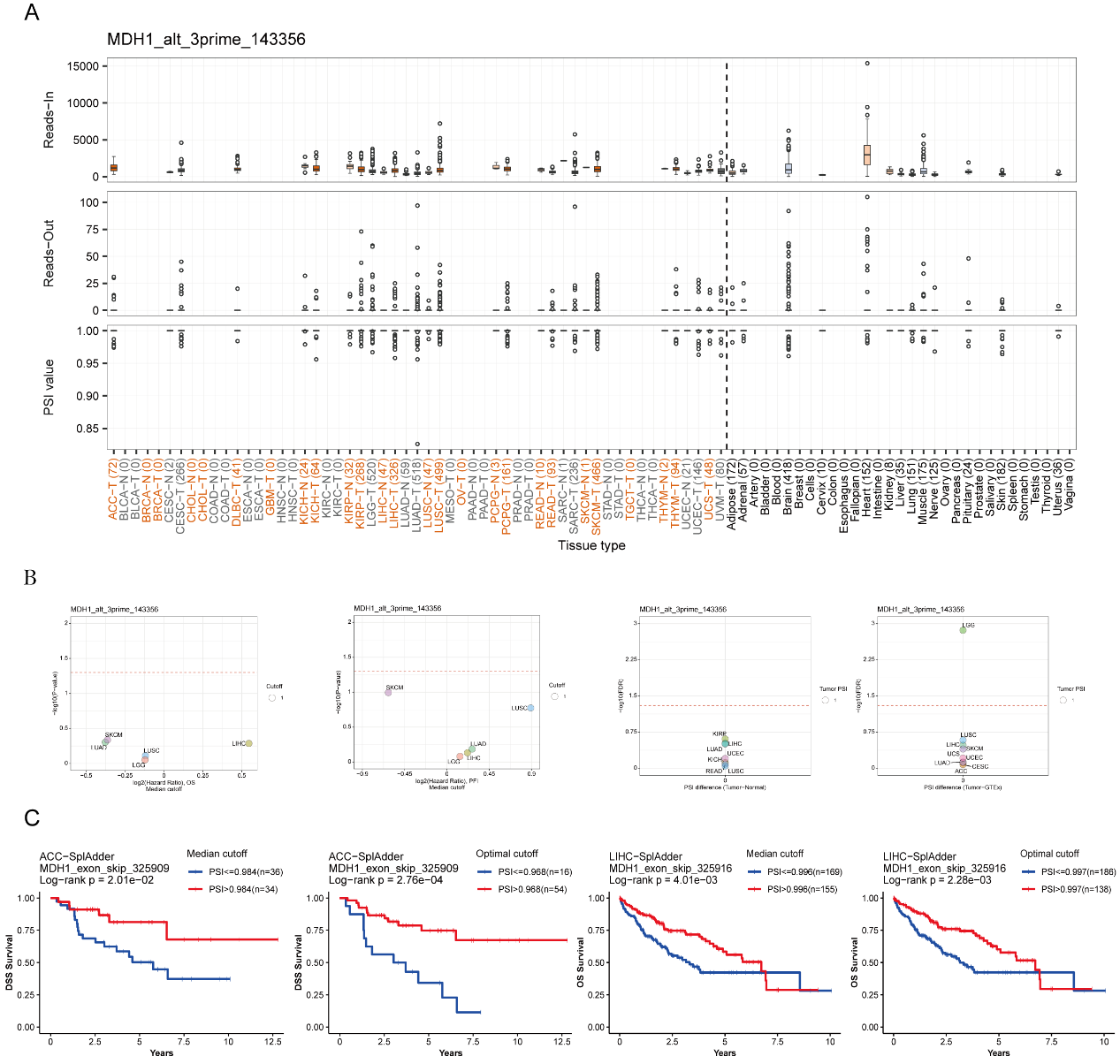


Supplementary Fig. 5

Alternative MDH1 splicing events. Reads-in, reads-out, and PSI values MDH1_.alt_3prime_143356 in pan-cancers, adjacent samples, and healthy tissue samples. Colored labels correspond to tumors and matching adjacent tissues, while non-tumor tissues are labeled in black (A). PSI differences when comparing tumors and corresponding healthy or adjacent tissues and the association between MDH1_alt_3prime_143356 events and prognosis (B). K–M curves (C). *P < 0.05, **P < 0.01, ***P < 0.001.


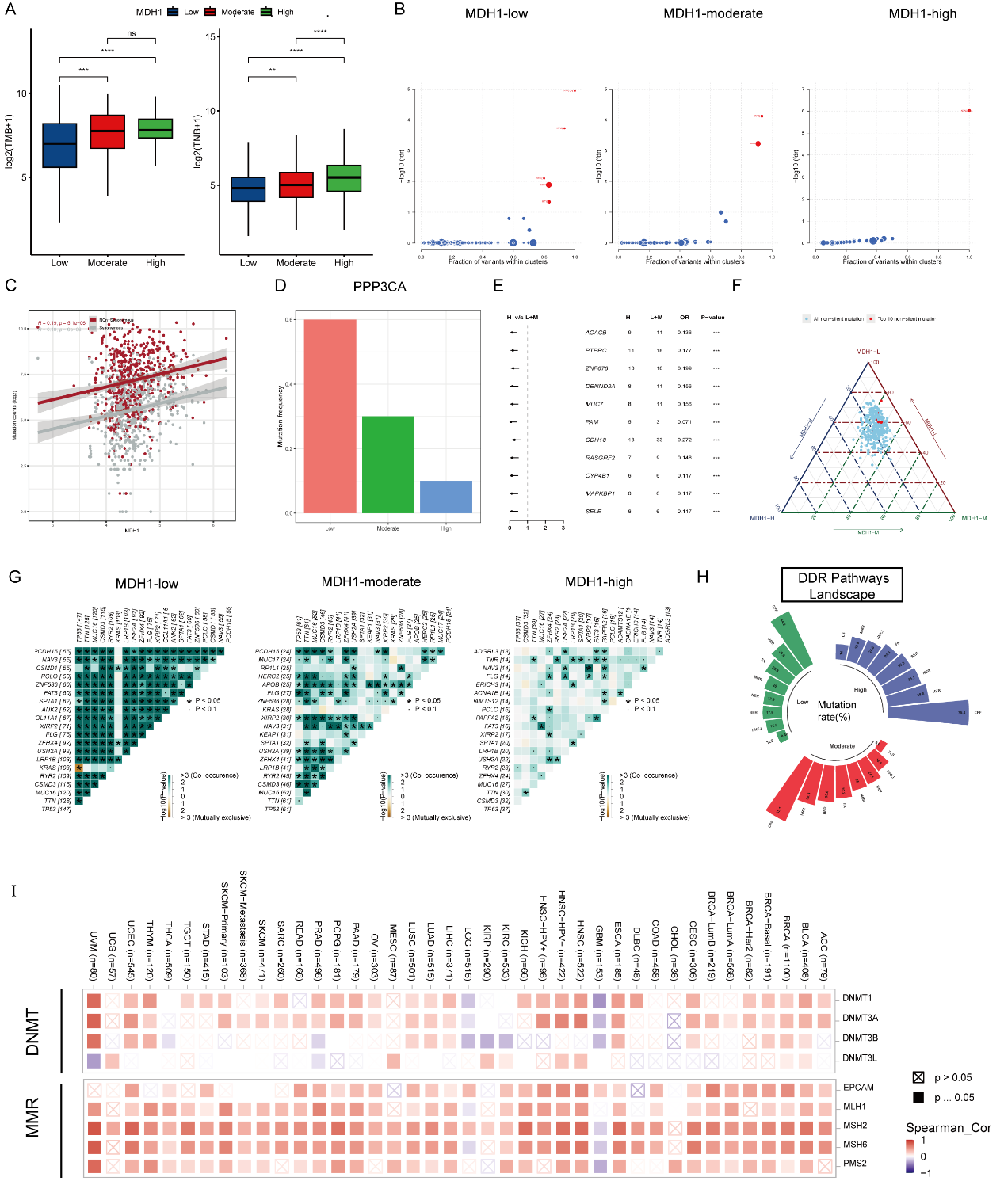
Supplementary Fig. 6

Association between MDH1 and DDR mutation frequency, intra-tumor heterogeneity and TMB in LUAD. TMB and TNB scores(A). PPP3CA is a mutual driver gene in both MDH1-L and MDH1-M groups, while no driver gene was detected in the MDH1-H group(B). Correlation between MDH1 expression and non-synonymous and synonymous mutations(C). PPP3CA mutation frequency(D). Significantly mutated genes were identified in the MDH1-L+M group compared to the MDH1-H group, and ACACB, PTPRC, and ZNF676 are the three most frequently mutated genes in MDH1-L+M samples. *** p < 0.001(E). A ternary diagram was plotted to depict the distribution of all non-silent mutations in the three MDH1 groups, and the 10 most frequent non-silent mutations (TP53, AMT, PRKDC, FANCM, BRCA2, POLE, HFM1, MSH4, ATR, and BRIP1) were highlighted in red dots(F). Mutually exclusive mutations and co-occurrence (G). Seven DDR pathways with mutation rates(H). Correlation analysis between MDH1 and DNMT, MMR(I).


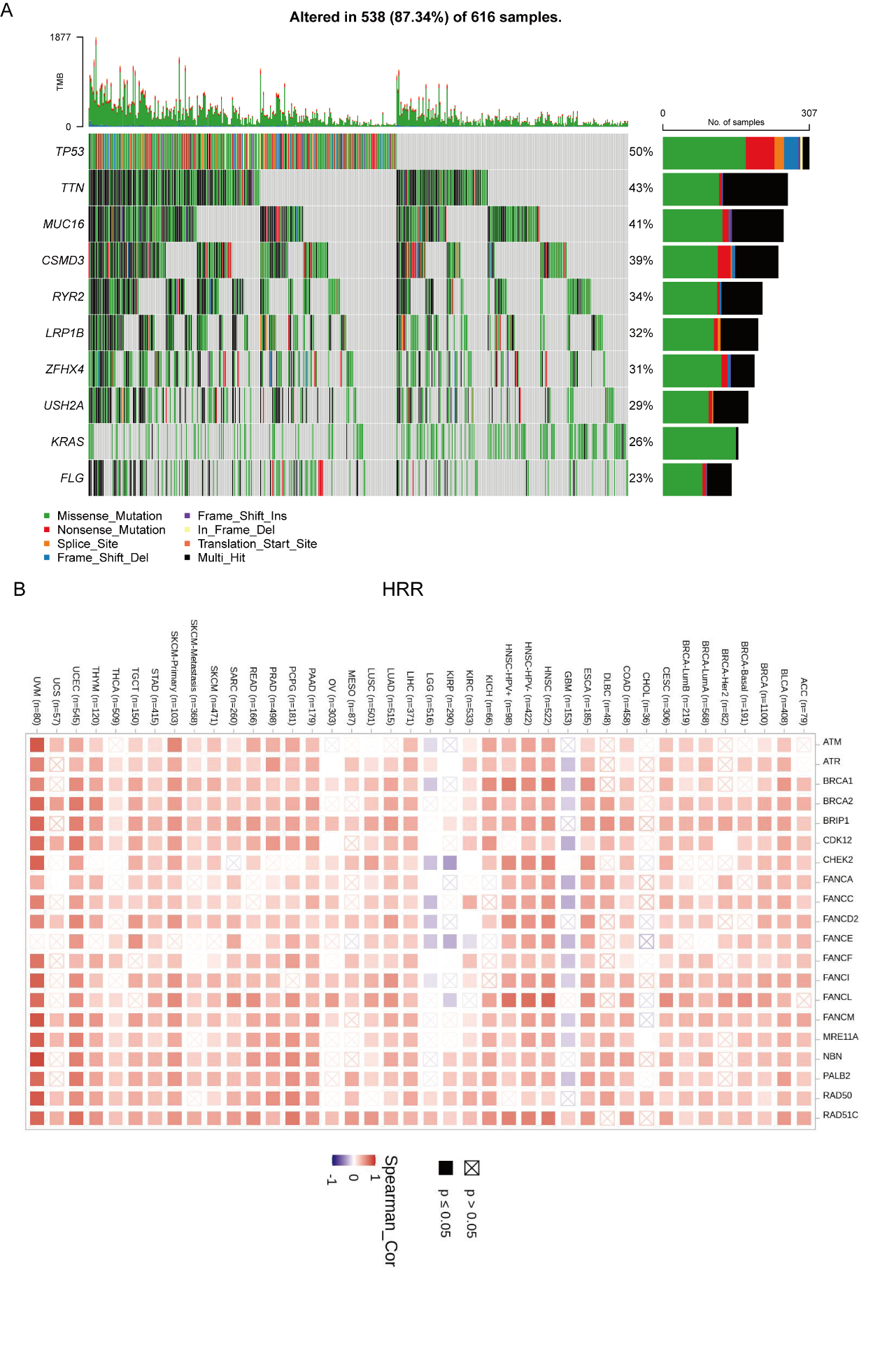


Supplementary Fig. 7

An oncoplot shows the 10 most frequent non-silent mutations in LUAD(A). The correlation analysis based on HRR related genes(B).


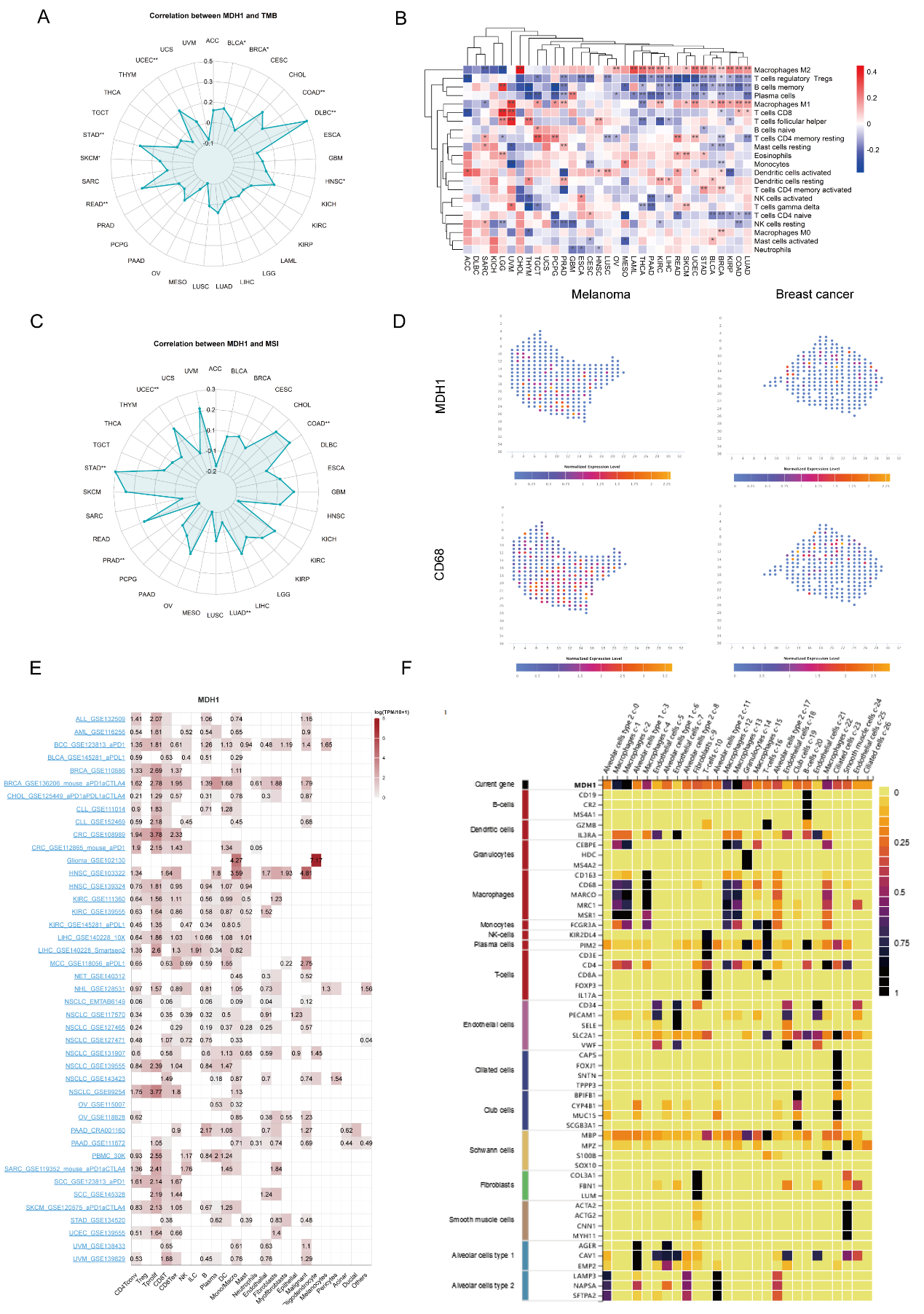


Supplementary Fig. 8

The role of MDH1 in immune microenvironment. TMB (A), MSI (C). The heatmap of association between MDH1 and various immune cells in pan-cancer based on CIBERSORT calculation(B). Spatial transcriptome analysis in spatialDB(D). single analysis of MDH1 in TISCH(E) and HPA(F).


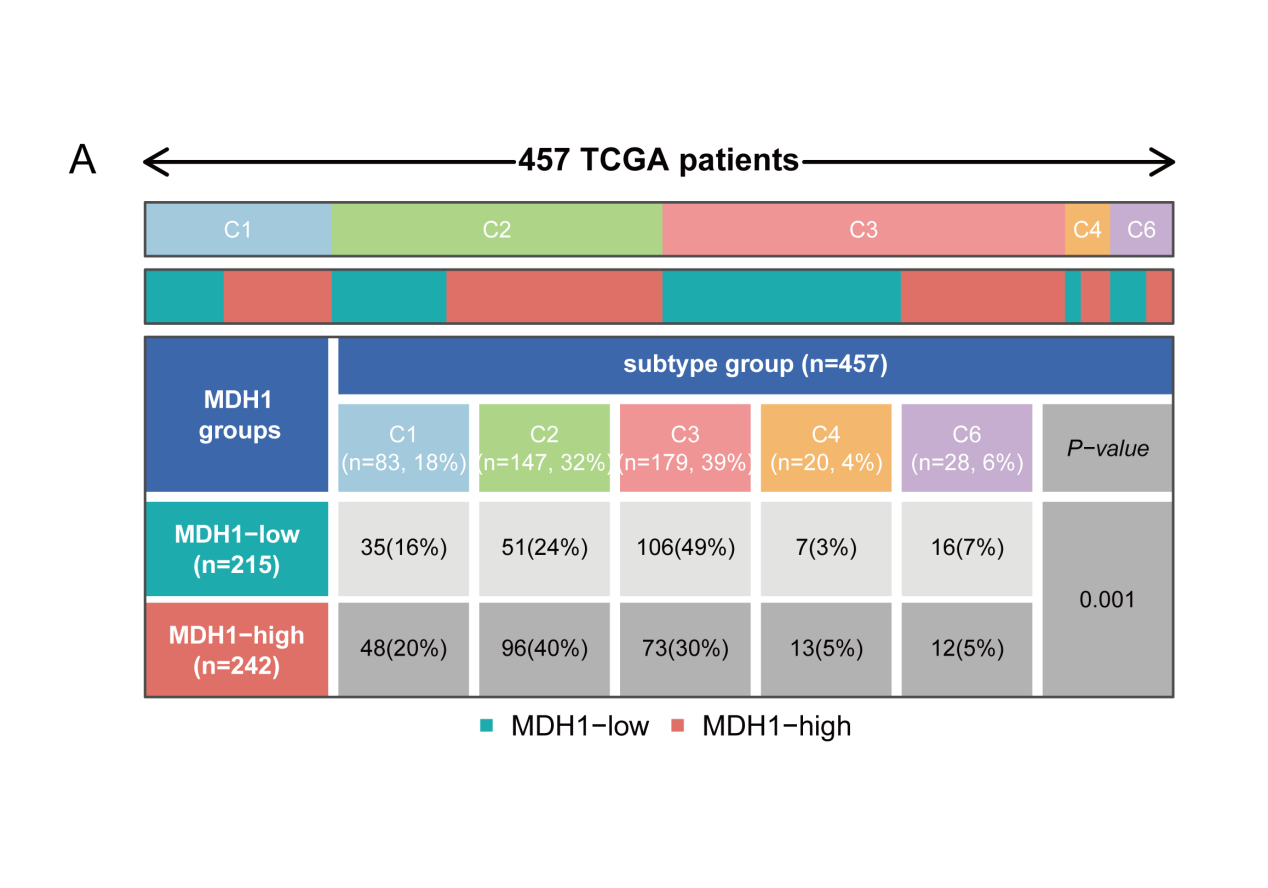


Supplementary Fig. 9

Heatmap and table illustrating the distribution of the TCGA immune clusters in the MDH1 sets(A).


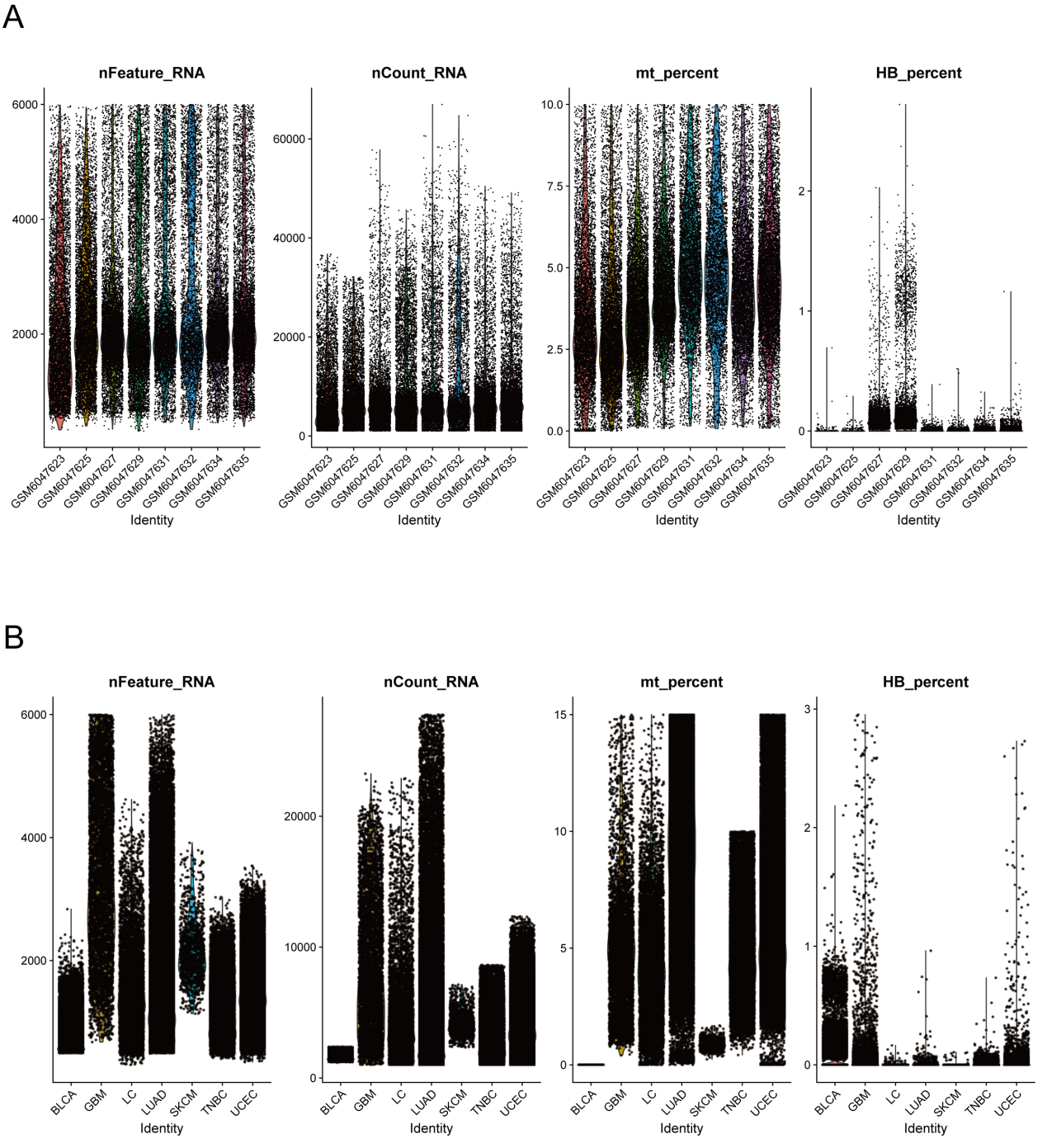


Supplementary Fig. 10

Quality control of single-cell data. ScRNA-LUAD cohort(A). Pan-cancer immunotherapy scRNA(B).


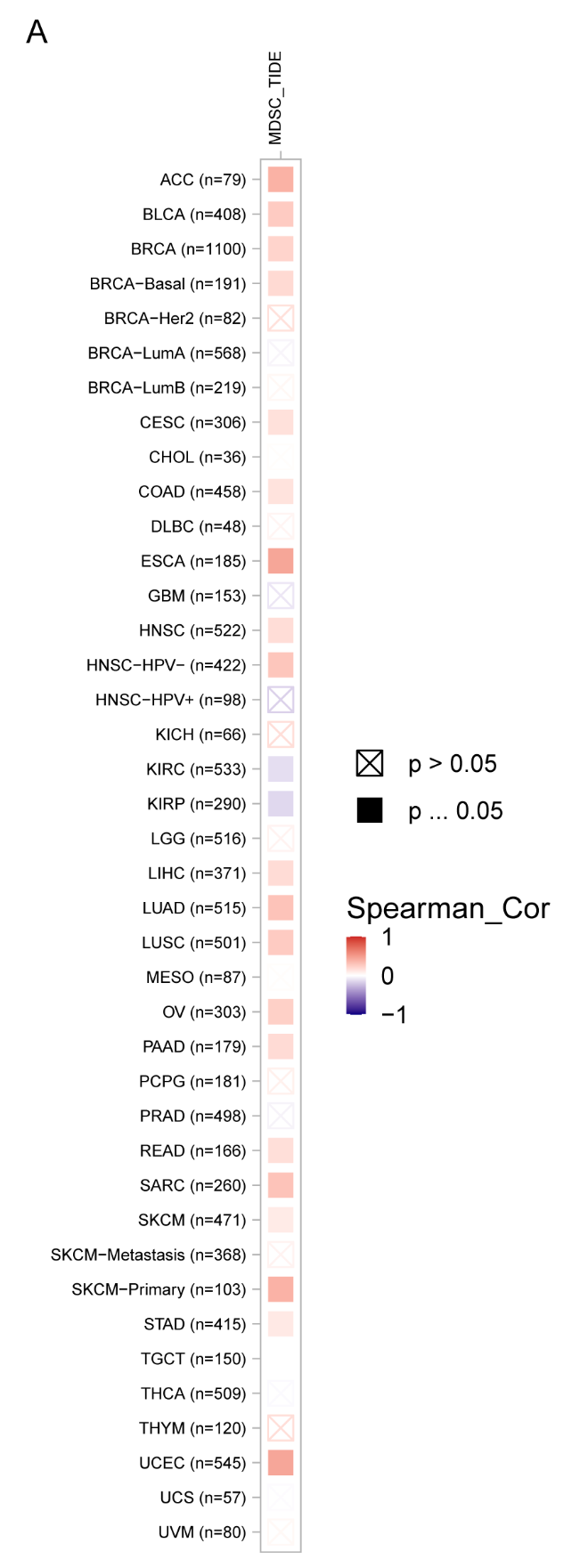


Supplementary Fig. 11

The correlation analysis based on MDSC levels in pan-cancer cohort(A).


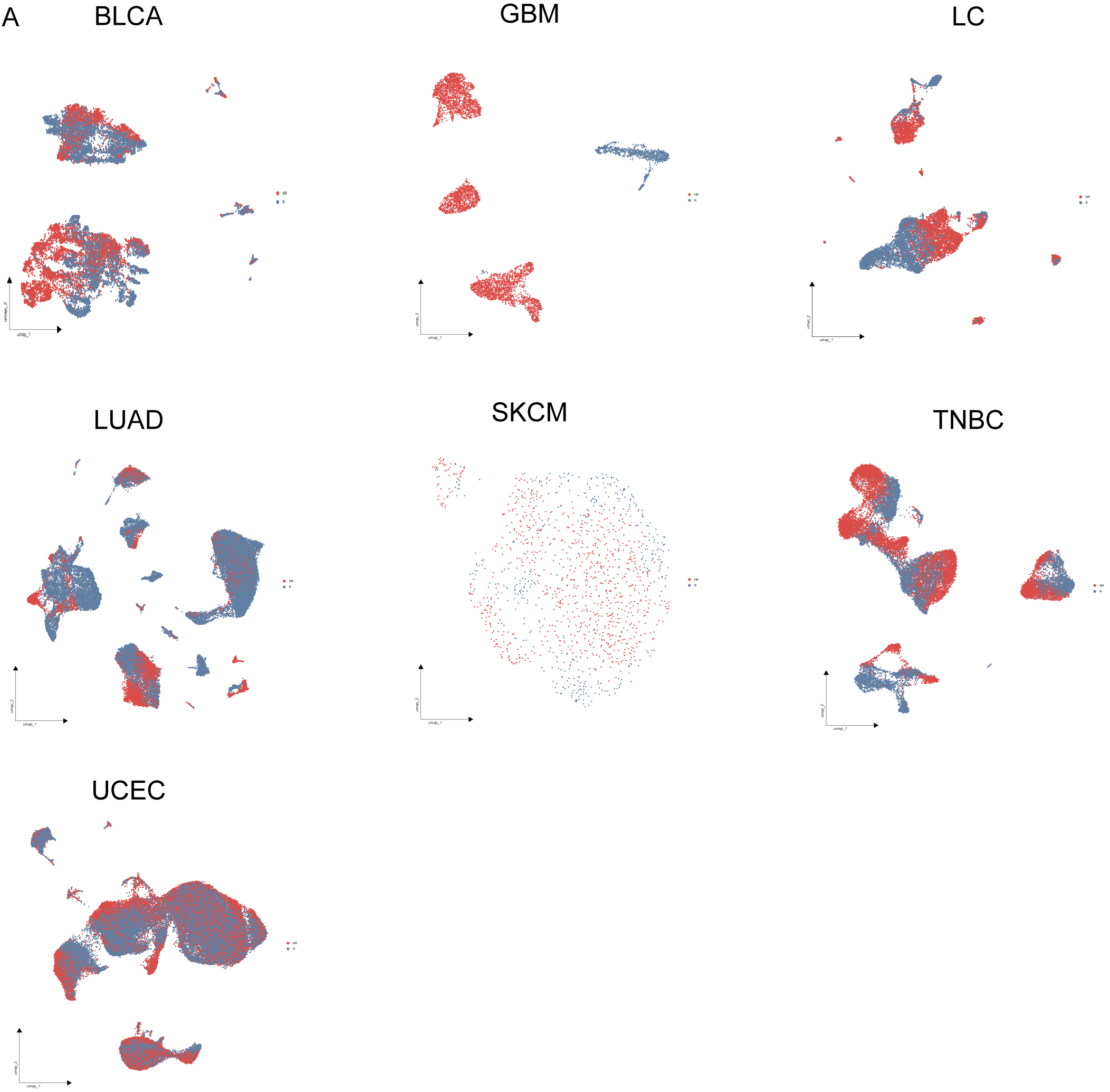


Supplementary Fig. 12

UMAP of immunotherapy responsiveness across cancer types (A).


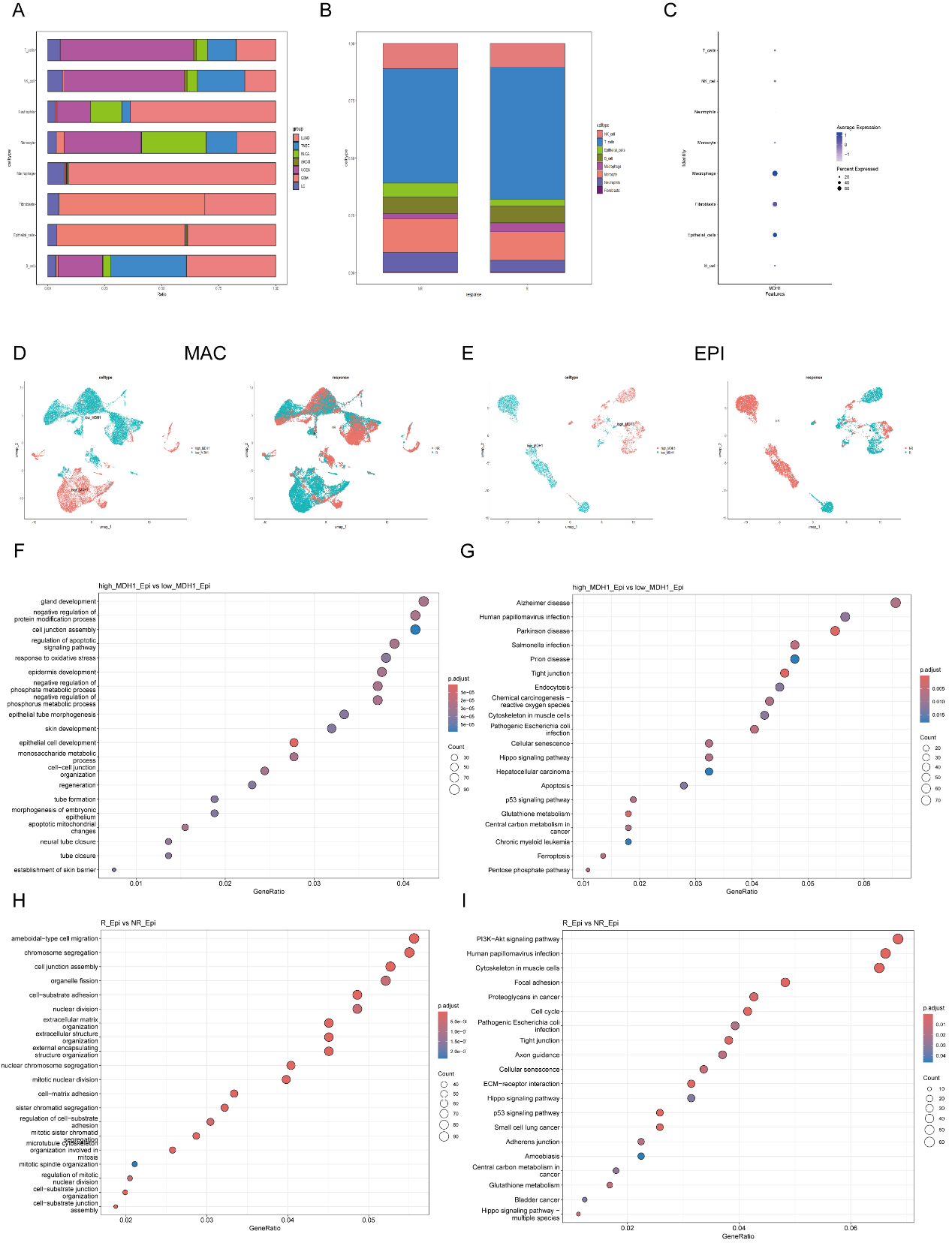


Supplementary Fig. 13

The proportion chart of celltypes(A) and immunotherapy responsiveness(B) in each cancer cohort. The correlation MDH1 and celltypes(C). The umap of macrophage subgroup(D), epithelial cell subgroup(E). In epithelial cell subgroup, the GO and KEGG analysis in MDH1-high and -low group(F-G), the GO and KEGG analysis in R and NR group(H-I).


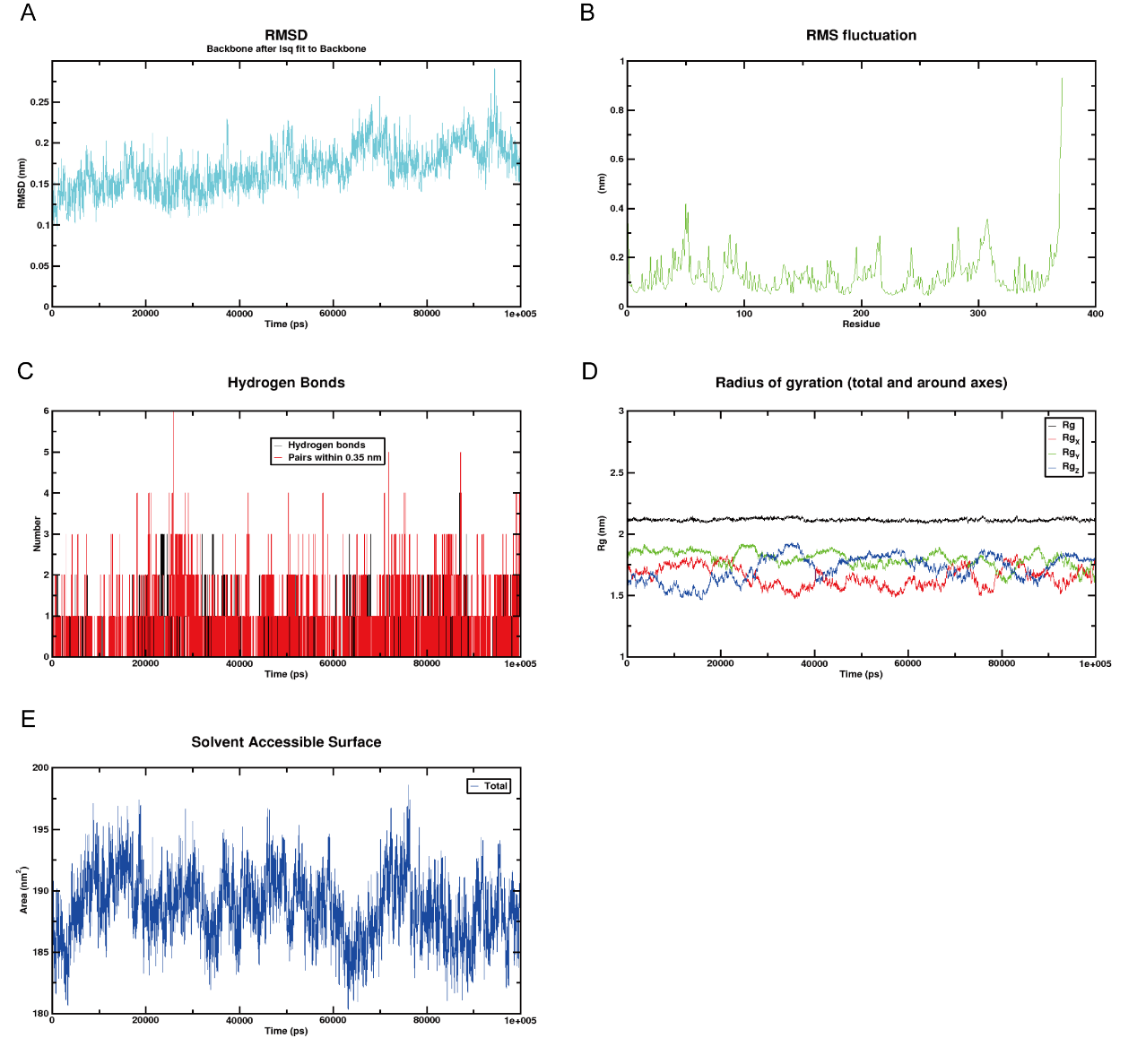


Supplementary Fig. 14

Molecular dynamic simulation analysis based on GROMACS. RMSD(A), RMSF(B), hydrogen bonds(C), gyration(D), solvent accessible surface(E).


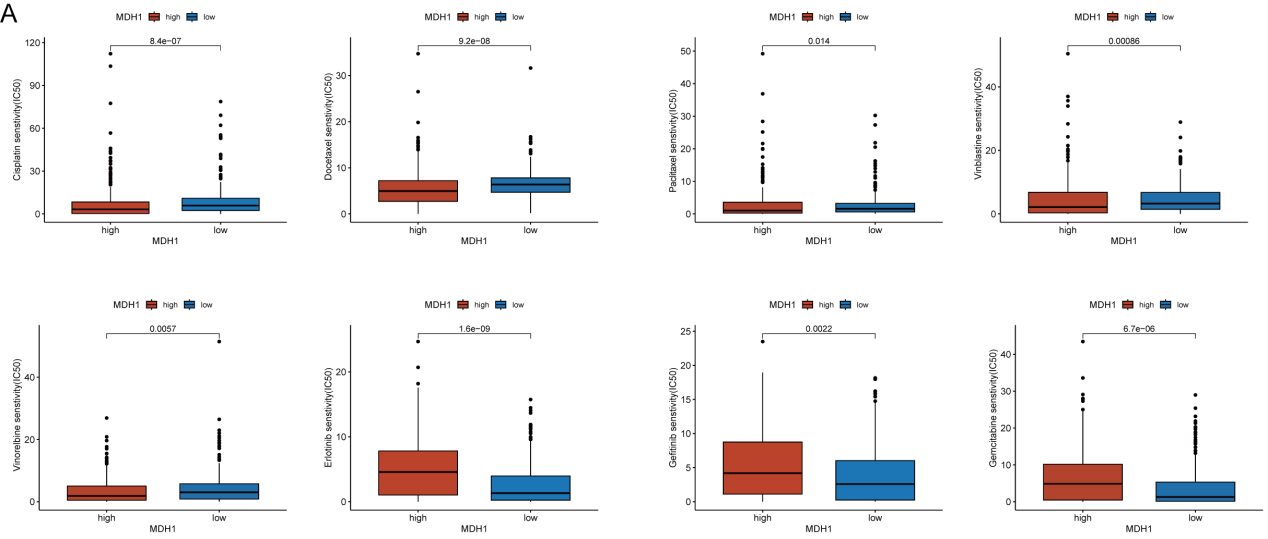


Supplementary Fig. 15

Relationships between the MDH1 and common chemotherapeutic sensitivity (A).


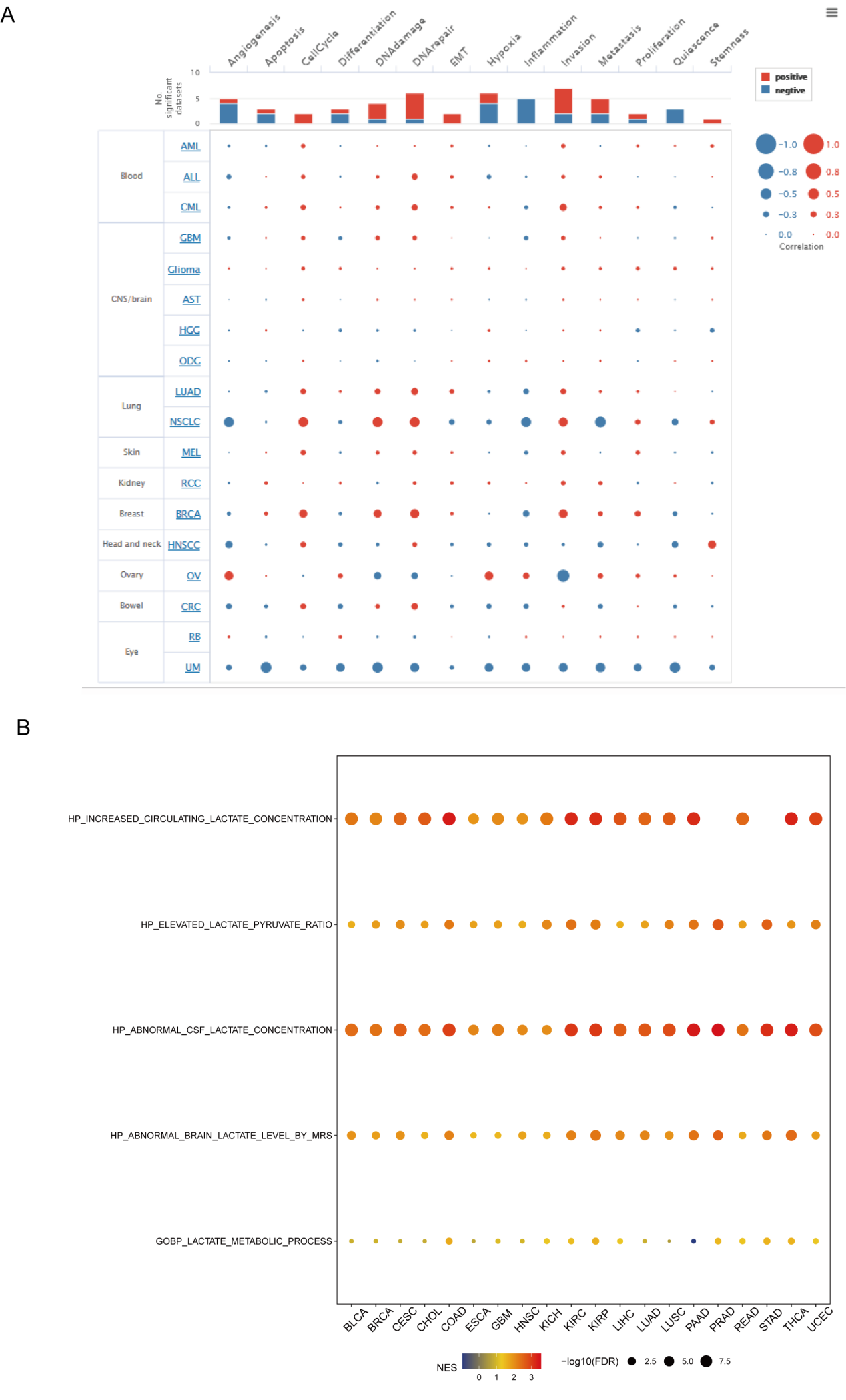


Supplementary Fig. 16

Functional enrichment based on MDH1 in a pan-cancer cohort. The heatmap from cancerSEA(A). The correlation analysis based on Lactate metabolism-related pathways(B).


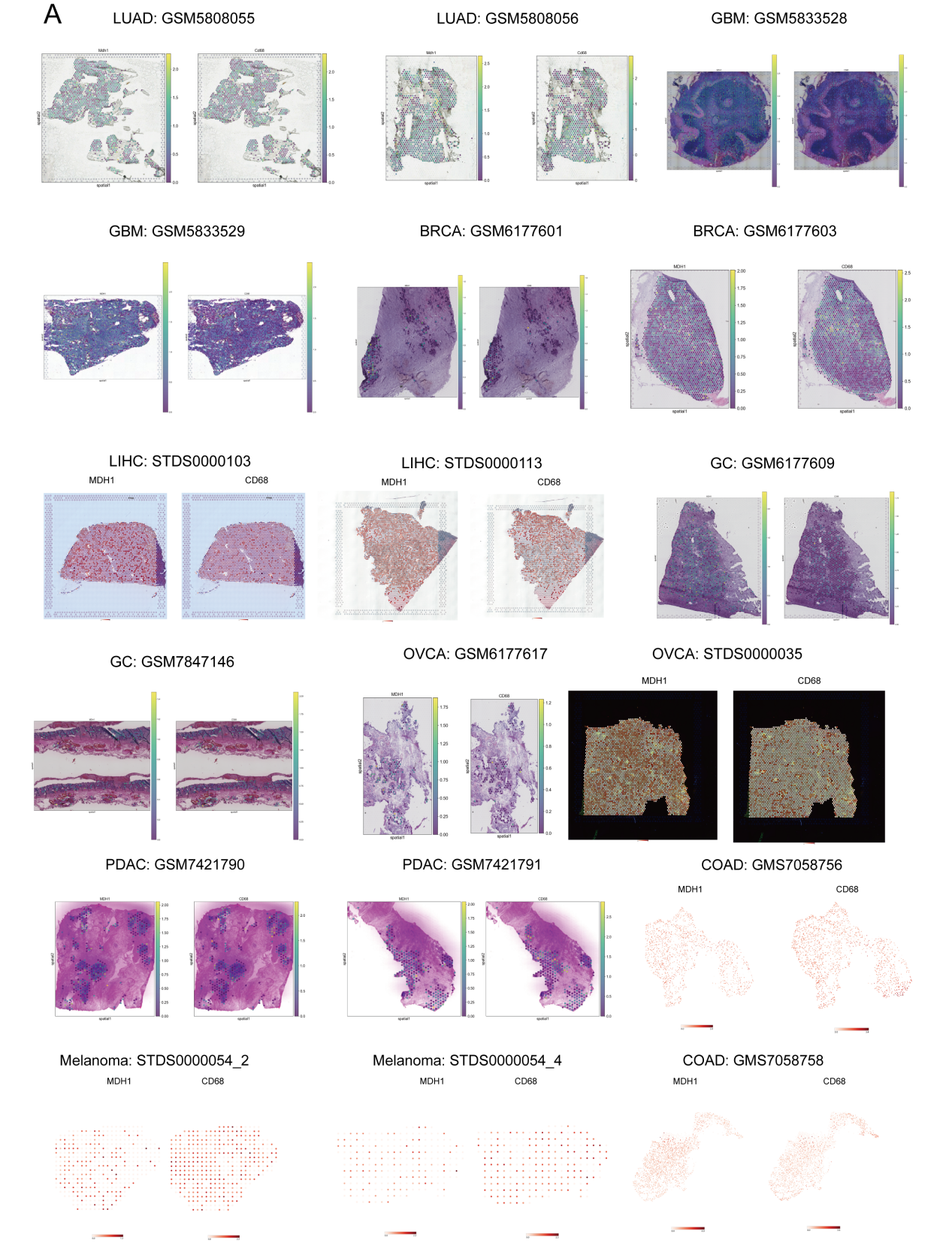


Supplementary Fig. 17

The pan-cancer spatial transcriptome cohort. The co-localization analysis based on MDH1 and CD68 expression(A).


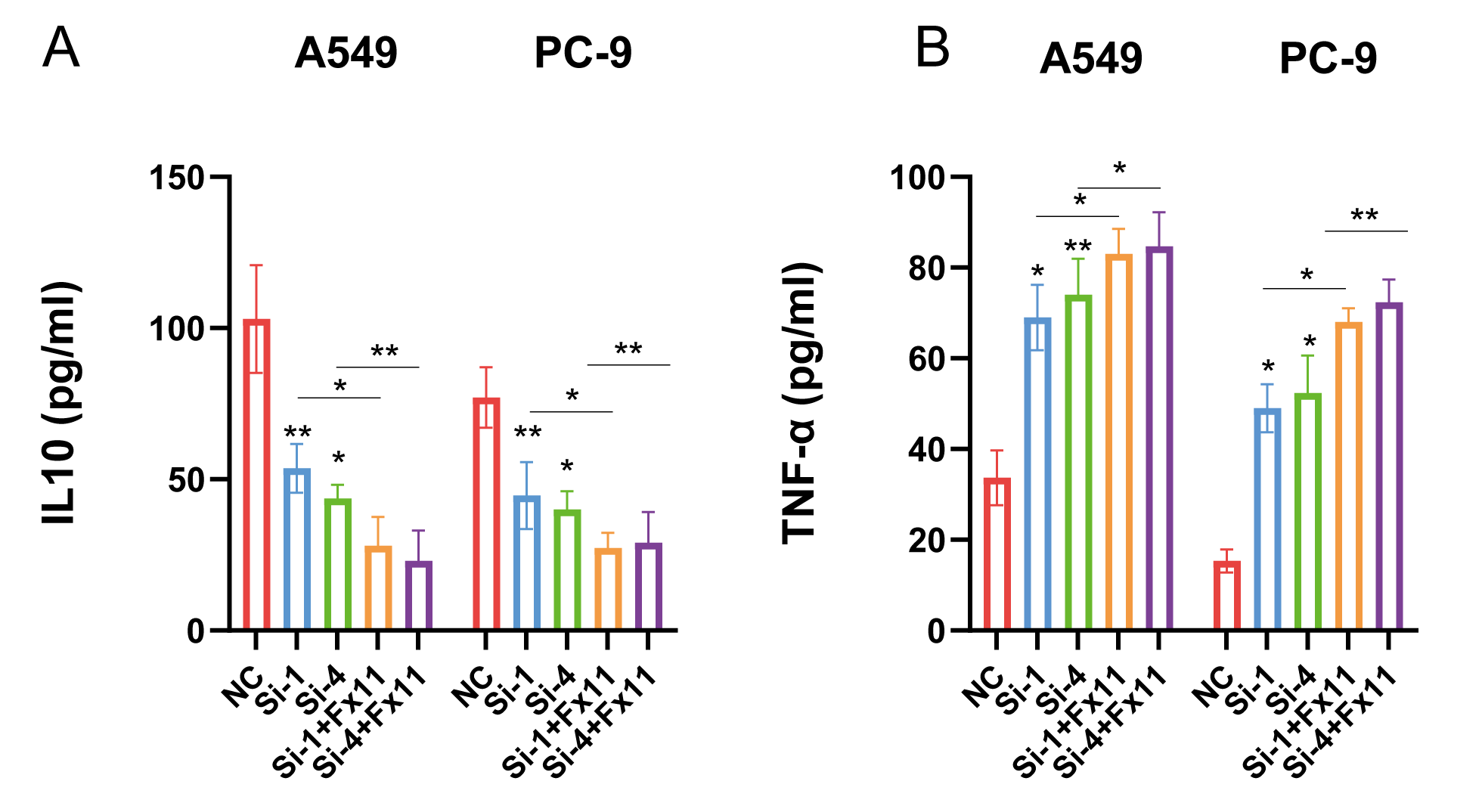


Supplementary Fig. 18

Knockdown of MDH1 suppressed the expression of IL10 (A) and THF-α (B) in LUAD cells by ELISA. LUAD cells were exposed to either vehicle or the LDHA antagonist FX11 at 10 mM for 24 h.


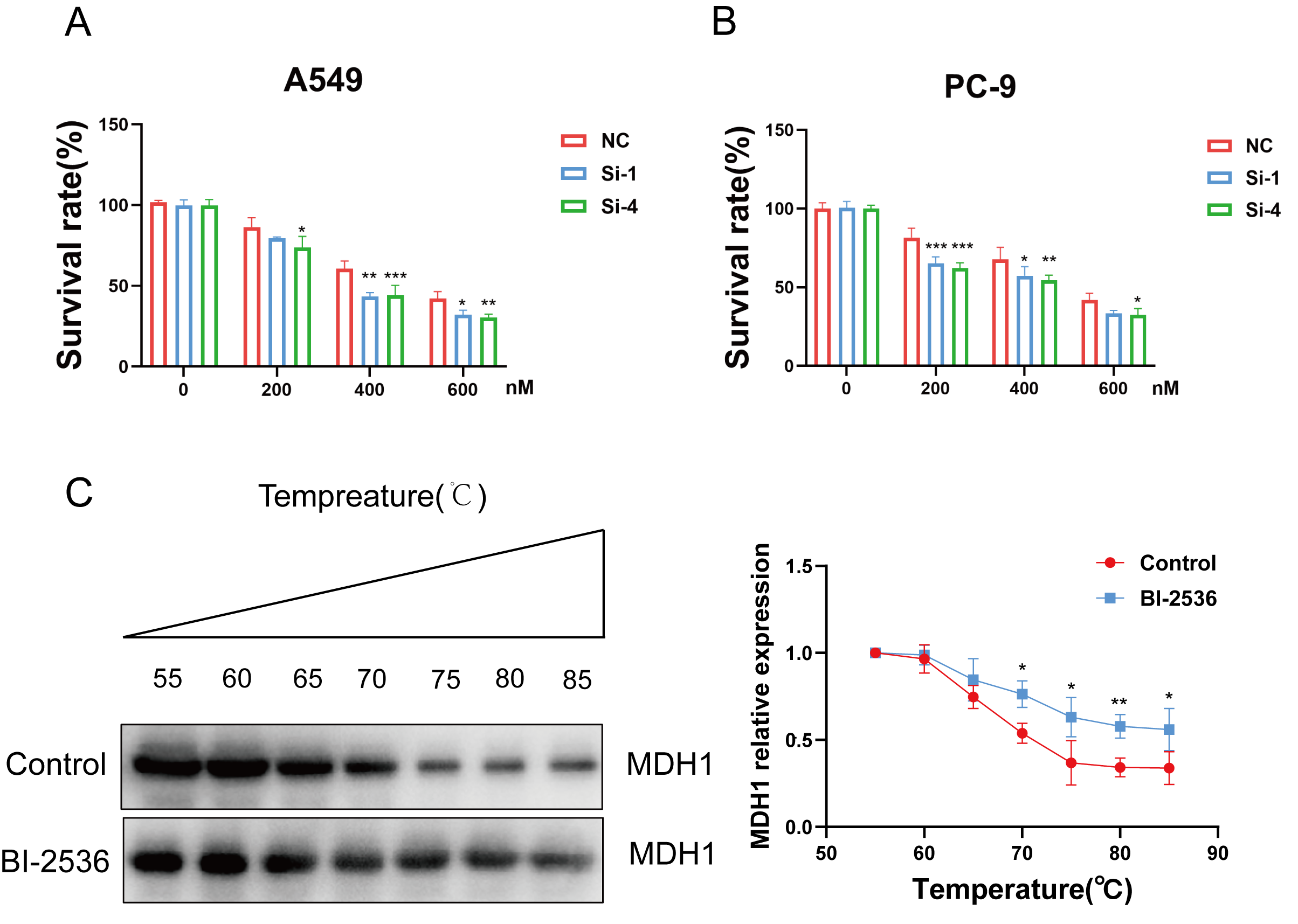


Supplementary Fig. 19

BI-2536 impedes growth in LUAD cells. A549(A), PC-9(B). Cellular thermal shift assay (CETSA) of MDH1 with BI-2536 in A549 cell(C).
